# INTS10–INTS13–INTS14 form a functional module of Integrator that binds nucleic acids and the cleavage module

**DOI:** 10.1038/s41467-020-17232-2

**Published:** 2020-07-09

**Authors:** Kevin Sabath, Melanie L. Stäubli, Sabrina Marti, Alexander Leitner, Murielle Moes, Stefanie Jonas

**Affiliations:** 1 Institute of Molecular Biology and Biophysics, ETH Zurich, Otto-Stern-Weg 5, CH-8093 Zurich, Switzerland; 2 Institute of Molecular Systems Biology, ETH Zurich, Zurich, Switzerland

**Keywords:** RNA, Non-coding RNAs, Transcription, X-ray crystallography

## Abstract

The Integrator complex processes 3′-ends of spliceosomal small nuclear RNAs (snRNAs). Furthermore, it regulates transcription of protein coding genes by terminating transcription after unstable pausing. The molecular basis for Integrator’s functions remains obscure. Here, we show that INTS10, Asunder/INTS13 and INTS14 form a separable, functional Integrator module. The structure of INTS13-INTS14 reveals a strongly entwined complex with a unique chain interlink. Unexpected structural homology to the Ku70-Ku80 DNA repair complex suggests nucleic acid affinity. Indeed, the module displays affinity for DNA and RNA but prefers RNA hairpins. While the module plays an accessory role in snRNA maturation, it has a stronger influence on transcription termination after pausing. Asunder/INTS13 directly binds Integrator’s cleavage module via a conserved C-terminal motif that is involved in snRNA processing and required for spermatogenesis. Collectively, our data establish INTS10-INTS13-INTS14 as a nucleic acid-binding module and suggest that it brings cleavage module and target transcripts into proximity.

## Abstract (web summary)

The Integrator complex (INT) is responsible for the 3′-end processing of several classes of non-coding RNAs. Here the authors show that the INTS10-INTS13-INTS14 complex forms a distinct submodule of INT and suggest it facilitates RNA substrate targeting.

## Introduction

RNA polymerase II (RNAPII) requires essential processing factors to terminate transcription and to release its primary transcripts via endonucleolytic cleavage^1^. For several indispensable short non-coding RNA transcripts in metazoans, this initial 3′-end processing step is carried out by the Integrator complex (INT)^2,3^. INT was first identified as the long sought-after 3′-end processing complex for uridine-rich small nuclear RNAs (UsnRNAs), which form the center of the splicing machinery^4^. Later, it was also shown to cleave nascent transcripts of enhancer RNAs (eRNAs), which are transcriptional regulators in metazoans^5^, telomerase RNA^6^, a simian viral micro-RNA precursor (pre-miR HSUR4)^7^ and spliced leader snRNAs in nematodes^8^.

Recently, evidence has been accumulating from studies in *Drosophila* that suggest a more widespread role of INT in transcription regulation of protein coding genes. INT promotes transcription termination after unstable RNAPII pausing via cleavage of nascent transcripts^9,10^. Furthermore, INT has been reported to contribute to RNAPII initiation, pause release, and termination on protein-coding genes^11–13^. Due to its important functions, depletion of individual INT subunits (INTS) is lethal during embryonic development in all organisms tested so far^14–17^ and the complex is implicated in numerous diseases^18^. However, the molecular mechanism of INT recruitment, specificity and action in these processes is poorly understood.

INT associates with the C-terminal domain (CTD) of RNAPII in the presence of phosphorylation marks on Ser7 and Ser2 in consecutive CTD heptapeptide repeats^19,20^, consistent with its recruitment to transcripts of <400 nucleotides^21^. INT presence on UsnRNA genes furthermore requires their gene-specific promoter structure consisting of a distal and proximal sequence element (DSE/PSE) together with transcription factors Oct-1, Sp1, and SNAPc^22^. In addition, cleavage of the nascent transcript depends on a sequence element (GTTTN_0-3_AAARNNAGA) downstream of the UsnRNA 3′-processing site, which has been termed 3′-box^23^. INT processing is also required for faithful RNAPII termination on UsnRNA genes, since its depletion or inhibition of its CTD-binding leads to transcriptional read-through^24,25^.

INT is absent from lower eukaryotes such as yeast but conserved in higher eukaryotes^26^. It consists of at least 14 subunits in human cells together amounting to ~1.5 MDa^27–29^. For most subunits, function and structure remain uncharacterized. A notable exception is INTS11 that has been identified as the active endonuclease of the complex early on^4,30^, based on sequence homology with the metallo-β-lactamase of the mRNA 3′-end processing machinery^31^. INTS11 contains all active site residues required for hydrolytic cleavage of RNA and forms a heterodimer with an inactive paralog, the pseudo-enzyme INTS9^32,33^.

Instead of existing as a monolithic holo-complex, evidence from several studies suggests that INT might assemble in a stepwise manner from separate modules^2,3^: For example, the catalytic heterodimer INTS9–INTS11 was shown to copurify with INTS4 from nuclear cell extracts in a lower molecular weight peak distinct from the holo-complex, suggesting that these three subunits form the cleavage module of INT^4,34^. Consistently, targeted ChIP analyses of several INTS on U2 snRNA suggest that INTS11 joins the core of the complex only towards the 3′-end^20^. Recently, evidence for a potential second INT module was reported in a study that described a role for INTS13 in enhancer activation during cell differentiation^35^: In size exclusion chromatography of nuclear extracts, INTS13 not only co-migrated with the INT holo-complex but also appeared in a second, lower molecular weight peak. In INTS13 immunoprecipitations (IP) from this second peak, two additional INTS (INTS10, INTS14) were detected by mass spectrometry (MS).

*INTS13* was initially named *Mat89Bb*^36^ and later *Asunder*^37^. It was characterized as an essential factor for embryonic development in *Drosophila* and *Xenopus* and shown to be required for correct mitosis in human cells^36,38,39^. Mutation of *INTS13* leads to sterile male flies, indicating an important regulatory role during spermatogenesis^37^. More recently, INTS13 has been shown to be indispensable for human cell differentiation^35^. These diverse cellular outcomes of *INTS13* mutation/depletion could potentially be downstream results of impaired INT involvement in a broad-range of RNAPII transcription events. However, studies differ on whether they suggest that INTS13 functions within the INT complex^13,39^, or that it has additional roles outside of the complex^35^.

Here, we show that human INTS13, INTS14, and INTS10 form a stable functional entity and characterize this new INT module biochemically, structurally, and functionally. We map the interaction network between the three proteins in cells and in vitro, and show that it has low micromolar affinity for DNA and RNA. The crystal structure of the INTS13–INTS14 heterodimer reveals that the chains of the two proteins are physically interlinked. Furthermore, the INTS10–INTS13–INTS14 subcomplex directly binds the cleavage module, consisting of INTS4–INTS9–INTS11. This interaction is mediated by the INTS13 C-terminus that has previously been shown to be required for spermatogenesis in flies, suggesting that cleavage module binding is the molecular basis for INTS13′s role in cell differentiation. Reporter assays demonstrate that INTS13 binding to the cleavage module is also required for efficient UsnRNA 3′-end processing in human cells. Collectively, the data suggest that the INTS10–INTS13–INTS14 subcomplex forms a nucleic acid (NA)-binding module of INT that helps to bring the cleavage module into proximity of transcripts.

## Results

### INTS13 and INTS14 form a pseudo symmetric heterodimer

**Fig. 1:**
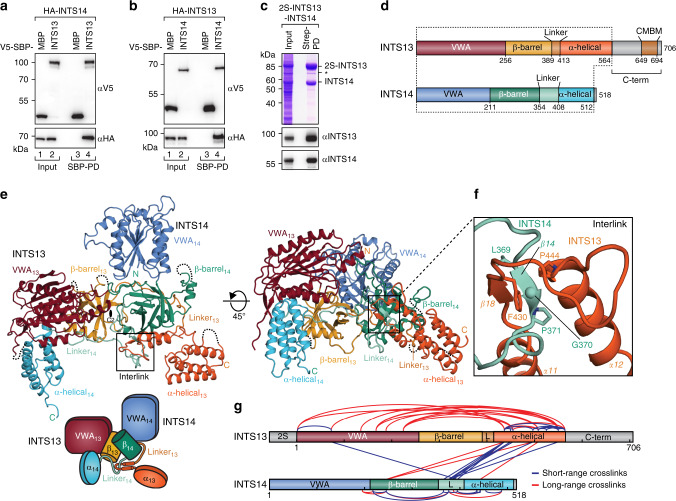
INTS13 and INTS14 form an interlinked heterodimer. **a** Coprecipitation experiments of λN-HA-tagged INTS14 with V5-SBP-tagged INTS13 from HEK293T cells using streptavidin beads. V5-SBP-MBP served as a negative control. Inputs (αV5-blot 1%, αHA-blot 0.38%) and bound fractions (αV5-blot 7.5%, αHA-blot 20%) were analyzed by Western blotting. **b** Same experiment as in **a** but with λN-HA-INTS13 and V5-SBP-INTS14. **c** Copurification of INTS14 with 2S-tagged INTS13 from insect cells expressing both proteins. The identity of proteins on the Coomassie-stained gel was verified by Western blotting. The asterisk marks a C-terminal degradation product of INTS13. C-terminal truncations of INTS13 are not detected by the specific antibody. **d** Domain organization of INTS13 and INTS14 as observed in the crystal structure of the complex. The dotted line marks portions of both proteins that are visible in the structure. CMBM marks the cleavage module-binding motif identified in this study. **e** Structure of the INTS13–INTS14 complex colored as in **d**. The two-fold rotation axis (C2) for the pseudosymmetry is indicated by a black oval. The position of the chain interlink is marked by a black box. The cartoon provides a simplified schematic for the domain arrangement of the complex. **f** Close-up of the interlink between INTS13 (dark orange) and INTS14 (light blue). **g** DSS crosslinks between Lys residues of INTS13 and INTS14 as determined by XL–MS. Crosslinks between residues that are visible in the crystal structure are shown, including crosslinks within the INTS13–INTS14 complex (blue) and crosslinks stemming most likely from complex oligomerization (red). Domain schemes are colored as in **d** and every 100th residue is marked with a tick. L indicates linker regions.

In order to test whether human (*Hs*) INTS13 and INTS14 interact with each other, we first performed copurification assays from HEK293T cells overexpressing both proteins. We found that V5-streptavidin-binding peptide (SBP)-tagged INTS13 specifically coprecipitated hemagglutinin (HA)-tagged INTS14, and conversely V5-SBP tagged INTS14 coprecipitated HA-INTS13 (Fig. 1a, b). We repeated the pulldown from insect cells that coexpressed tandem Strep-tagged (2S) *Hs*INTS13 and untagged *Hs*INTS14 after baculovirus infection. It verified that INTS13 and INTS14 form a stoichiometric complex (Fig. 1c), that also remained stable upon further purification (Supplementary Fig. 1a).

To elucidate the molecular basis of their interaction and gain insight into their potential function, we crystallized the INTS13–INTS14 complex and solved its structure to 2.5 Å resolution using a combination of multiple isomorphous replacement (MIR) and native single-wavelength anomalous diffraction (sulfur-SAD) (Supplementary Table 1). The structure reveals that both proteins share a surprisingly homologous domain organization and form an interlinked, strongly entwined, pseudosymmetric heterodimer (Fig. 1d–f).

Both INTS13 and INTS14 consist of an N-terminal von-Willebrand type A like domain (VWA), followed by a central β-barrel domain and a C-terminal α-helical domain that is connected by a long linker devoid of secondary structure elements (Fig. 1d). This striking structural homology could not have been predicted based on the low sequence similarity of the two proteins (12% identity, 21% similarity). In both proteins, only a few surface loops that face away from the dimer interface and several residues at the termini are disordered (see “Methods” section). In addition, ~140 residues from the mainly unstructured INTS13 C-terminus are missing from the structure (residues 565–706). Judging from the double band we observe for INTS13, its C-terminus gets progressively degraded upon expression and purification of the protein complex (Supplementary Fig. 1a).

**Fig. 2:**
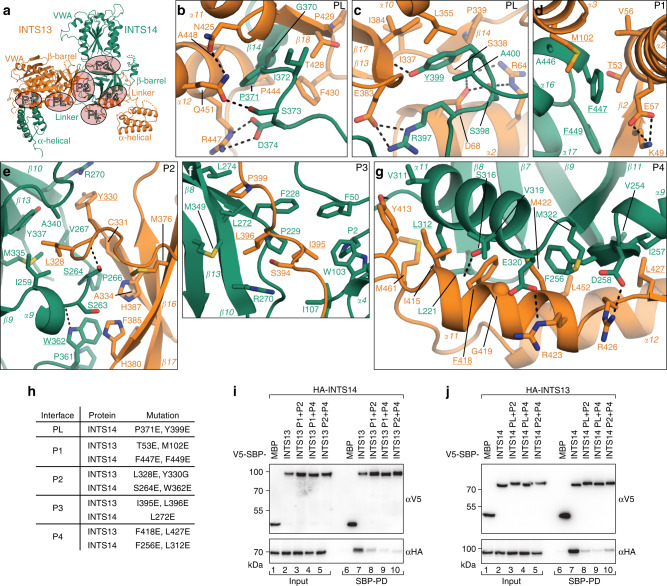
The extensive interface between INTS13 and INTS14 requires several mutations for disruption. **a** Overview of the complex between INTS13 (orange) and INTS14 (green) with interface patches PL and P1-4 highlighted by orange ovals and labeled accordingly. **b**–**g** Close-up views of the interface patches PL and P1–4 as indicated in **a**. Secondary structure elements as well as side-chains of interface residues are shown and labeled. Dotted lines indicate hydrogen bonds. Residues mutated in this study are underlined. **h** List of point mutations in interface patches that were tested for effects on complex formation. **i**, **j** Coprecipitation experiments from HEK293T cells expressing patch double mutants in V5-SBP-INTS13 **i** or INTS14 **j** with the corresponding λN-HA-tagged binding partner. V5-SBP-MBP and the wt proteins were used as negative and positive controls, respectively. Inputs (αV5-blots 1%, αHA-blots 0.5%) and bound fractions (αV5-blots 4%, αHA-blots 20% and 10%) were analyzed by Western blotting.

The interface of the INTS13–INTS14 complex is extensive (11,180 Å^2^) and involves all domains of both proteins. Most of the interaction surfaces are hydrophobic and framed by polar hydrogen bonds or salt bridges (Fig. 2b–g, Supplementary Figs. 2, 3). At the center of the complex, the two homologous β-barrel domains form a major contact around a rotational pseudo-symmetry axis (Fig. 1e). Another large and complex interface is generated by the interdomain linkers of INTS13 and INTS14, which fold onto the surface of the respective opposite β-barrels and also contact the VWA domains. Both linkers are well ordered in the electron density, consistent with the extensive, mainly hydrophobic contacts they make with the folded domains (Supplementary Fig. 1c). In addition, the α-helical C-termini contribute to binding by packing against the β-barrel (INTS13) or VWA domain (INTS14) of their respective binding partner.

### The chains of INTS13 and INTS14 are interlinked

A particularly remarkable and unusual feature of the interface is formed by the linker region of INTS14, which threads through a loop between the first two α-helices (α8, α9) of the α-helical domain of INTS13, thereby generating a physical interlock of the two protein chains (Figs. 1f and 2b). The INTS14 linker is overall highly conserved despite its length and lack of secondary structure elements (Supplementary Fig. 3). In particular, the residues that pass through the INTS13 loop, an LG(PISD)-motif (*Hs*INTS14 369–374), are maintained throughout metazoans. Conversely, INTS13 loop residues LxPF (*Hs*INTS13 427–430), which contact the LG-motif in our structure, are only conserved in metazoan genomes where also INTS14 is present (INTS14 is absent in nematodes, Supplementary Fig. 3). Together, this strong sequence conservation suggests that the unusual interlinked conformation is present throughout all metazoans that express both INTS13 and INTS14.

Formation of such an interlinked dimer is difficult to rationalize from already mature, folded proteins, and suggests that in cells INTS13/INTS14 folding and dimerization are coupled. This observation might also explain why recombinant expression of the individual proteins led to insoluble aggregates, while soluble proteins were obtained upon coexpression of both subunits.

To probe whether the conformation that we observed in the crystal is also present in solution, we performed crosslinking coupled to mass spectrometry (XL–MS) (Fig. 1g, Supplementary Fig. 4a–c and Supplementary Data 1). Disuccinimidyl suberate (DSS) was used to covalently link lysine residues with a distance between C_α_ atoms below 26–30 Å^40^. Importantly, several crosslinks between the INTS13 and INTS14-linker regions around the interlink site (INTS13 Lys 436, 449, 454, and INTS14 Lys 365, 376) support a close spatial proximity of these two regions in solution as expected from the crystal structure. Furthermore, several crosslinks between the INTS14 linker and the INTS13 α-helical or VWA domains confirm that their spatial arrangement is maintained in solution.

In addition, several long-range crosslinks were detected between the VWA and α-helical domains of INTS13. These residues lie on opposite sides of the protein and therefore suggest oligomerization of the INTS13–INTS14 complex in solution. Indeed, the purified complex elutes from a size exclusion column as double peak in fractions that would correspond to the molecular weight of a dimer and a trimer of the complex (*M*_w,monomer_ = 143 kDa, *M*_w,dimer_ = 287 kDa, *M*_w,trimer_ = 429 kDa, Supplementary Fig. 4d). Consistent with oligomer formation also in cells, V5-SBP-tagged INTS13 coprecipitates HA-tagged INTS13 from human cells and the same is true for INTS14 in the analogous experiment (Supplementary Fig. 4e, f).

In summary, the data corroborate that the crystal structure constitutes a faithful representation of the INTS13–INTS14 complex in solution and further suggest that it oligomerizes in cells.

### Multiple mutations are required to dissociate INTS13–INTS14

To further verify the arrangement that we observed in the crystal structure, we designed point mutants along the INTS13–INTS14 interface and tested their effect on complex formation in coprecipitation assays from HEK293T cells (Fig. 2h–j, and Supplementary Figs. 1–3). Given the strongly intertwined heterodimerization of INTS13–INTS14, we predicted that disruption of single contact points would not suffice to prevent binding. Thus, we identified four main interaction patches (P1–P4, Fig. 2a), which we mutated alone or in different combinations in INTS13 and INTS14: Patch 1 lies between INTS13 VWA and INTS14 α-helical domain, patch 2 between the two β-barrels, patch 3 connects INTS13 linker and INTS14 β-barrel, and patch 4 is on the interface of INTS13 α-helical domain and INTS14 β-barrel. Furthermore, we introduced amino acid substitutions in the linker of INTS14 (PL) to prevent interlock formation (Fig. 2b, c, h, and Supplementary Fig. 3).

As expected from the structure, mutation of single patches in V5-SBP-tagged INTS13 led to no (P3) or only small decreases (P1, P2, P4) in binding affinity compared to wild type (wt, Supplementary Fig. 1d). Similarly, substitutions in the analogous patches of V5-SBP-tagged INST14 showed only slight reductions in copurification of HA-INTS13 compared to wt (P1–3, Supplementary Fig. 1e). In contrast, introduction of mutations in the interlocking linker of INTS14 or the adjacent P4 patch strongly decreased interactions with overexpressed HA-INTS13 (Supplementary Fig. 1e, lanes 11 and 14).

Efficient disruption of the INTS13–INTS14 complex could only be achieved by combining substitutions in two interaction patches (Fig. 2i, j). Consistent with a multi-surface interaction network, combining mutations in P1 and P2 of INTS13 led to an additive loss of copurification efficiency (Supplementary Fig. 1d lanes 9 and 10 vs. Fig. 2i lane 8). Furthermore, mutations in either INTS13 or INTS14 targeting the interlink site (P4 or PL) in combination with another patch almost completely abolished heterodimerization (Fig. 2i lanes 9 and 10, Fig. 2j lanes 8–10).

Together, these results support the strongly intertwined heterodimer that we observed in the crystal structure and underpin the importance of interlinking for INTS13–INTS14 complex formation.

### INTS13 and INTS14 form a stable module with INTS10

Previously, INTS10 was shown to co-IP with INTS13 from nuclear extracts of HL-60 cells^35^. We could recapitulate this interaction in HEK293T cells, where V5-SBP-tagged INTS13 efficiently copurified both endogenous INTS14 and INTS10 (Supplementary Fig. 5a). This interaction is direct, since both INTS10 and INTS14 copurified in stoichiometric amounts with 2S-tagged INTS13 after recombinant expression from insect cells. The complex remained stably associated during two additional purification steps (Supplementary Fig. 5b), indicating that INTS13–INTS14 and INTS10 bind each other with high affinity.

**Fig. 3:**
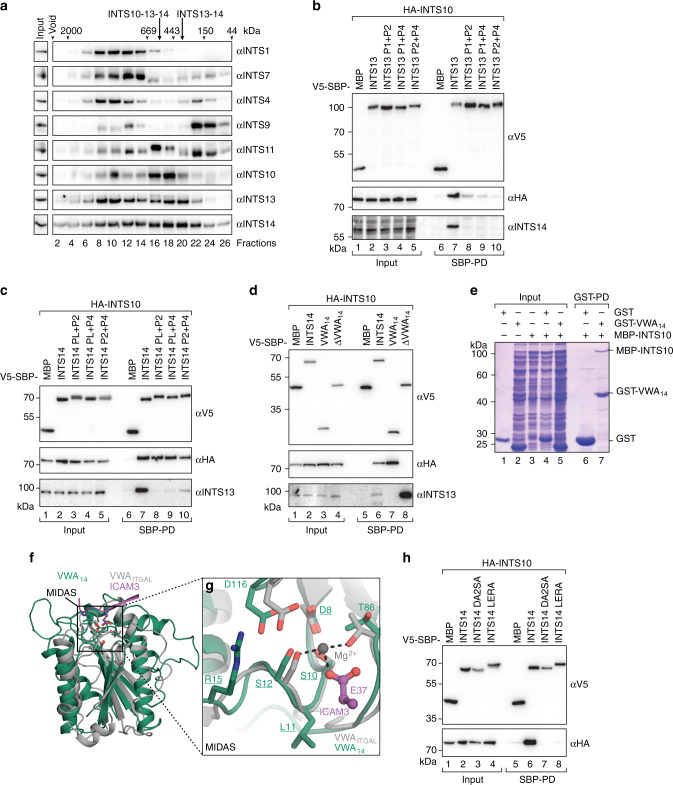
INTS10 forms a module with INTS13–INTS14 in cells and binds to the MIDAS pocket of the INTS14 VWA domain. **a** Size exclusion chromatography of nuclear extract from HEK293T cells. All even fractions were analyzed by Western blotting. INTS1 and INTS7 served as marker proteins for the INT core, while INTS4–9–11 form the cleavage module. Elution points of molecular weight markers are indicated on the top. In addition, elution volumes of purified INTS13–INTS14 and INTS10–INTS13–INTS14 are indicated. **b**, **c** Coprecipitation of HA-INTS10 from HEK293T cells with V5-SBP-INTS13 **b** or V5-SBP-INTS14 **c**. Wild-type and binding patch double mutants were compared. Coprecipitation of endogenous INTS14 or INTS13 is detected as control for complex disruption. Inputs (αV5-blots 1%, αHA-blots 0.38% and 0.75%, αINTS14-blot 0.38%, αINTS13-blot 0.03%) and bound fractions (αV5-blots 3%, αHA-blots 20 and 15%, αINTS14/αINTS13-blots 20%) were analyzed by Western blotting. **d** Deletion constructs of V5-SBP-INTS14 were tested for copurification of HA-INTS10 and endogenous INTS13. Inputs (αV5-blot 1%, αHA-blot 1%, αINTS13-blot 0.1%) and bound fractions (αV5-blot 4%, αHA-blot 5%, αINTS13-blot 20%) were characterized by Western blotting. **e** Copurification of MBP-INTS10 with GST-INTS14-VWA from *E. coli* lysates. GST served as negative control. **f** Structure superposition (r.m.s.d. 3.6 Å over 160 residues) of the VWA domain of INTS14 (green) with the VWA domain of integrin-αL (ITGAL, gray, PDB-ID: 1T0P^73^) in complex with intercellular adhesion molecule 3 (ICAM3, purple). The black square highlights the position of the MIDAS pocket, residues lining the pocket are shown as sticks. **g** Focused view of the MIDAS pockets of both VWA domains highlights conservation of the site in INTS14. The Mg^2+^-ion in the ITGAL pocket that coordinates a Glu of the interaction partner ICAM3 is shown as a gray sphere. Ion coordination is indicated by black dotted lines. Coloring as in **f**. Residues mutated in INTS14 are underlined. **h** Coprecipitation of HA-INTS10 from HEK293T cells with V5-SBP-INTS14 wt or MIDAS pocket mutants (D8A, S10A, S12A [DA2SA] and L11E, R15A [LERA]). V5-SBP-MBP served as control. Inputs (αV5-blots 1%, αHA-blots 0.75%) and bound fractions (αV5-blots 3%, αHA-blots 15%) were analyzed by Western blotting.

We next determined whether this trimeric complex also forms in the nucleus of human cells. Nuclear extract from HEK293T cells was fractionated by native complex size on a Superose 6 gel filtration column and analyzed by Western blotting against INT components (Fig. 3a). In addition to the INT holo-complex (fractions 8–10), INTS10, INTS13, and INTS14 comigrated in several fractions (fractions 16–18) that coincided with the elution volume of the recombinant trimeric INTS10–INTS13–INTS14 complex. These fractions did also not overlap with the peak of the cleavage module (fractions 22–24). This behavior suggests that the three proteins indeed form a discrete, modular component of INT in the nucleus of human cells that exists alongside the holo-complex and the cleavage module.

We used mutants that disrupt INTS13–INTS14 complex formation to identify which subunit binds INTS10. All INTS13 mutants that lost binding to endogenous INTS14 in pull-down experiments from HEK293T cells, simultaneously lost interaction with coexpressed HA-INTS10 (Fig. 3b) as well as endogenous INTS10 (Supplementary Fig. 5a). In contrast, all INTS14 mutants maintained the same level of INTS10 coprecipitation, despite their impaired INTS13 interaction (Fig. 3c). Thus, INTS14 provides the main interaction surface for INTS10 within the complex, consistent with previous yeast two-hybrid data for the *Dm*INTS10/INTS14 orthologs^27^.

Given that the VWA domain of INTS14 remains exposed in the complex and VWA domains are widely characterized as protein–protein interaction domains^41^, we hypothesized that it might form the INTS10-binding platform. Consistently, deletion of the INTS14 VWA domain (INTS14ΔVWA, residues 211–518) completely abrogates the INTS10 interaction in coprecipitation assays (Fig. 3d), while it does not impair binding to endogenous INTS13. Furthermore, the VWA domain by itself (VWA_14_, residues 1–210) is sufficient to recapitulate an even higher INTS10 pull-down efficiency than full length INTS14. Finally, recombinantly expressed GST-tagged INTS14 VWA specifically copurified MBP-INTS10 in vitro (Fig. 3d), demonstrating that INTS10 is integrated into the INTS13–INTS14 complex via the INTS14 VWA domain.

The surface of the INTS14 VWA displays a conserved patch that is located on top of the VWA domain (Supplementary Fig. 5c). This patch corresponds to the metal-ion-dependent adhesion site (MIDAS), a well-characterized ligand-binding pocket in VWA domains of several proteins such as integrins^42^, which mediates protein–protein interactions via a coordinated magnesium ion (Fig. 3f, g). Since the Mg^2+^-coordinating residues of this pocket are well conserved in INTS14, we hypothesized that INTS10 might bind via the MIDAS. Mutation of MIDAS residues (D8A, S10A, S12A [DA2SA] and L11E, R15A [LERA]) completely abrogated INTS10 coprecipitation with INTS14 from HEK293T cells (Fig. 3h), indicating that the MIDAS pocket of the INTS14 VWA makes a major contribution to the INTS10-binding interface.

Collectively, our data establish INTS10–INTS13–INTS14 as a new stable module of INT and suggest that INTS14 adopts a similar mode of binding to INTS10 as the one observed for cell surface receptors and their ligands.

### The INTS13–INTS14 complex shares homology with Ku70–Ku80

We performed a search with the DALI-server^43^ for proteins with similar structural features as INTS13 or INTS14 to obtain insight into their potential molecular function. Both Ku70 and Ku80 returned as the highest-scoring hits. These two proteins are required for repair of DNA-double-strand breaks by non-homologous end joining^44^. Despite low sequence similarities (identity 7–10%, similarity 17–19%), Ku70/Ku80 and INTS13/INTS14 share highly similar domain architectures with N-terminal VWA domains, central β-barrels and C-terminal α-helical domains (Supplementary Fig. 6a, b). In addition, Ku70–Ku80 form a pseudosymmetric heterodimer analogous to the INTS13–INTS14 complex (Supplementary Fig. 6c, d) and create a central tunnel for binding the end of a DNA double helix (Supplementary Fig. 6f). Superposition of the DNA from the Ku70–Ku80 structure onto INTS13–INTS14 results in extensive clashes (Supplementary Fig. 6e), indicating that the complex is unlikely to simply mirror the function of Ku70–Ku80. Nevertheless, given the highly analogous three-dimensional arrangement of the complex, we reasoned that the INT module might have a broadly similar role and bind NAs.

### The INTS10–13–14 module preferentially binds RNA hairpins

**Fig. 4:**
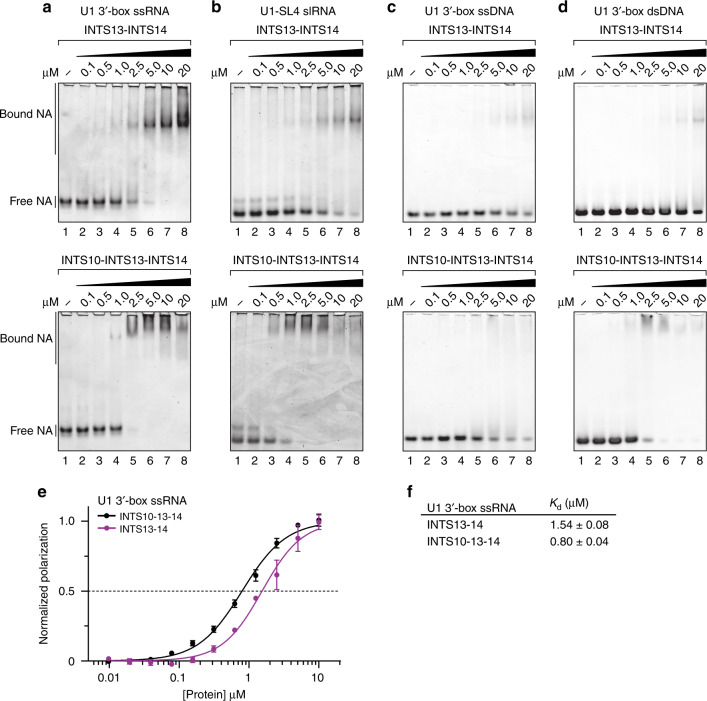
The INTS10–INTS13–INTS14 complex binds nucleic acids with a preference for stem loop RNAs. **a**–**d** EMSAs of INTS13–INTS14 (top panels) and INTS10–INTS13–INTS14 (lower panels) with U1 3′-box ssRNA **a**, U1-SL4 dsRNA **b**, U1 3′-box ssDNA **c**, and dsDNA **d**. Increasing protein concentrations were incubated with the respective nucleic acid, resolved on a native acrylamide gel and stained with a fluorophore. **e** Fluorescence polarization of FAM-labeled U1 3′-box ssRNA with INTS13–INTS14 (purple) and INTS10–INTS13–INTS14 (black), respectively. Error bars indicate standard deviations from the mean of triplicate experiments. **f** Dissociation constants with standard error of means derived from fitting data in **e**. Source data are provided as a Source Data file.

During transcription, INT encounters both single (ss) and double-stranded (ds) DNA as well as ssRNA and RNA stem loops (sl). Therefore, we systematically tested binding of these different NA species to heterodimeric INTS13–INTS14 or heterotrimeric INTS10–INTS13–INTS14 using electrophoretic mobility shift assays (EMSAs, Fig. 4a–d and Supplementary Fig. 6k). We used the sequence of the 3′-box of the U1 snRNA precursor for ssRNA, ssDNA, and dsDNA, and the U1 stem loop 4 (SL4) as well as pre-miR HSUR4 as the slRNA species. Both the dimeric and the trimeric complexes bind all tested types of NA, although in general the trimeric complex has higher affinity judging by the protein concentration that shifts ~50% RNA (Fig. 4a–d, upper vs. lower panels). RNA species were preferred over the corresponding DNA (Fig. 4a–d). While INTS13–INTS14 favored ssRNA (~5-fold), inclusion of INTS10 leads to a ~2-fold higher overall affinity for NA and a ~2-fold higher preference for slRNA vs. ssRNA. In EMSAs, the affinities of INTS10–INTS13–INTS14 for U1 SL4 or pre-miR-HSUR4 lie around ~1 µM.

To obtain a more quantitative picture of RNA binding in solution, we carried out fluorescence polarization (FP) assays (Fig. 4e, f and Supplementary Fig. 6g–j). Consistent with our observations in EMSAs, the trimeric complex bound 5′-fluorophore-labeled U1 3′-box ssRNA with ~2-fold higher affinity (*K*_d_ = 0.80 ± 0.04 µM) compared to the dimeric complex (*K*_d_ = 1.54 ± 0.08 µM). The complex has no sequence specificity for the 3′-box since similar affinities were measured with a poly-U RNA oligo (U_12_, *K*_d_ (INTS13–INTS14) = 0.57 ± 0.05 µM, *K*_d_ (INTS10–INTS13–INTS14) = 0.42 ± 0.02 µM), and furthermore U1 SL4 and pre-miR-HSUR4 were both bound with comparable affinities (Fig. 4b vs. Supplementary Fig. 6k). Consistent with this notion, ssRNA binding is strongly influenced by the salt concentration in the buffer with a low salt condition yielding ~7-fold higher affinities for the dimeric and ~18-fold for the trimeric complex. Together, this evidence speaks for a largely charge-mediated binding of the NA phosphate backbone by the INTS10–INTS13–INTS14 module.

Inclusion of INTS10 consistently increases NA affinity, however because INTS10 could not be expressed in isolation, and a structure of the trimeric complex could not be obtained, we cannot determine why this is the case. Nevertheless, our data clearly indicates that INTS10–INTS13–INTS14 is an INT module with general NA affinity and a preferential binding to slRNA.

### INTS13 binds the INT cleavage module via its C-terminus

**Fig. 5:**
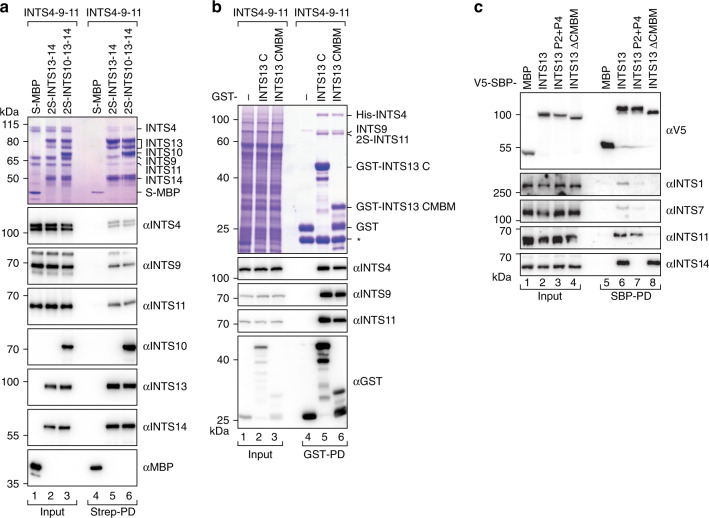
INTS10–INTS13–INTS14 binds the INT cleavage module via a conserved motif in the INTS13 C-terminus. **a** Coomassie-stained gel and corresponding Western blots from copurification of the INT cleavage module (INTS4–INTS9–INT11) with 2S-INTS13–INTS14 or 2S-INTS10–INTS13–INTS14 using purified complexes. S-MBP served as negative control. **b** Coomassie stained gel and corresponding Western blots from copurification of INT cleavage module from insect cell extracts with GST-INTS13 C-term constructs from *E.coli* lysates. GST served as negative control. The asterisk marks endogenous GST from insect cells that also binds to GSH-beads. **c** Coprecipitation of endogenous INT subunits with V5-SBP-INTS13 wild type and mutants. V5-SBP-MBP is included as a control. Inputs (αV5-blot 1%, αINTS14 0.5%, and others 0.1%) and bound fractions (αV5-blot 5%, αINTS14 10%, and others 20%) were analyzed by Western blotting.

We next sought to characterize how the module connects to other INTS. In copurification assays, both purified INTS13–INTS14 and INTS10–INTS13–INTS14 directly bound the purified cleavage module (INTS4–INTS9–INTS11), suggesting that binding is independent of INTS10 (Fig. 5a). Because INTS13 mutants that are deficient for INTS14-binding still coprecipitated endogenous INTS4, INTS9, and INTS11 from human cells (Supplementary Fig. 7a), we conclude that INTS13 provides a binding platform for the cleavage module. Since we observed in vitro that the INTS4–INTS9–INTS11 complex was not stoichiometrically co-eluted with INTS13–INTS14, and that INTS13 was C-terminally degraded (double band in Fig. 5a, lanes 2, 3, 5, and 6), we hypothesized that binding might be mediated by the C-terminus of INTS13. Indeed, the GST-tagged INTS13 C-terminus (INTS13 C, residues 566–708) strongly enriches all three recombinantly expressed cleavage module subunits in a pull-down from insect cells (Fig. 5b). Inspection of INTS13 sequence alignments and secondary structure predictions revealed a short, conserved α-helical section within the otherwise less conserved INTS13 C (residues 649–694, Supplementary Fig. 2). GST-pull down with this minimal construct resulted in equivalent enrichment of the cleavage module subunits, suggesting that these conserved C-terminal helices are sufficient for binding to the INT cleavage module, and thus we termed this section the cleavage module-binding motif (CMBM, Fig. 1d and Supplementary Fig. 2).

In the next step, we tested which subunit of the cleavage module is bound by CMBM in copurification assays, where each of the three subunits was expressed individually or in combinations with the other components (Supplementary Fig. 7b). Interestingly, we could only detect interaction with GST-CMBM if the full cleavage module was coexpressed. In addition, XL–MS of INTS13 CMBM in complex with INTS4–INTS9–INTS11 showed two crosslinks to the N-terminus of INTS4 and three to the C-terminal half of INTS11 (Supplementary Fig. 7c and Supplementary Data 2). Given that INTS4 is thought to use its N- and C-termini to clamp the heterodimeric INTS9 CTD–INTS11 CTD complex^33,34^, these observations suggest that INTS13 CMBM binds to a composite surface that is only present within the fully assembled cleavage module.

Finally, we used coprecipitations with INTS13 mutants to understand the connection of both INT modules to the remainder of the INT complex (INT core, Figs. 5c and 7c). Disrupting INTS13 binding to INTS14 weakens interaction with endogenous INTS (INTS1, INTS7), suggesting that the module is connected to the INT core via the INTS14–INTS10 axis. However, interactions with core subunits were lost completely if the CMBM of INTS13 was deleted (INTS13 ΔCMBM, residues 1–648), demonstrating that the cleavage module provides a stronger link to the INT core.

In summary, our observations suggest that the INTS10–INTS13–INTS14 NA-binding module could help to assemble the remainder of INT on target transcripts by binding the cleavage module using a conserved α-helical motif in the INTS13 C-terminus.

### INTS10–INTS13–INTS14 act together in UsnRNA processing

**Fig. 6:**
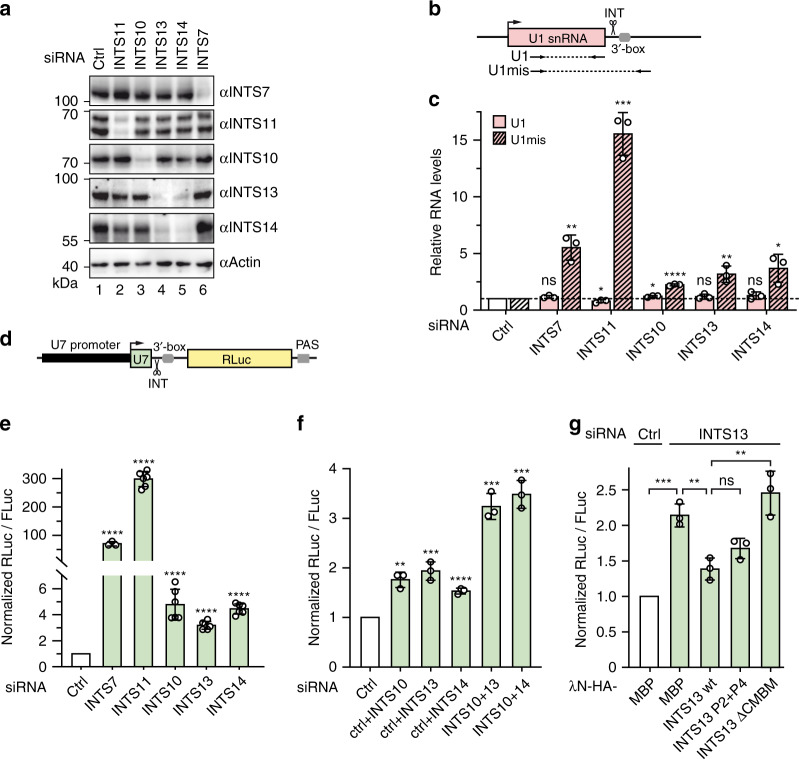
The INTS10–INTS13–INTS14 module has an accessory role in UsnRNA processing. **a** Representative Western blot of cells treated with siRNAs against INT subunits or non-targeting siRNA (corresponding to Fig. 7b). Actin served as loading control. **b** Scheme of qPCR-binding sites for amplification of mature (U1) and misprocessed (U1mis) U1 snRNA. **c** Relative U1 snRNA levels as determined by RT-qPCR. Clear bars represent U1 levels, while shaded bars represent levels of U1mis. All values were determined relative to U6 snRNA, which as an RNAPIII transcript is independent of INT, and were further normalized to the control knockdown condition. Statistical significance of U1 or U1mis levels was determined relative to their respective levels in the control depletion. **d** Scheme of U7 luciferase reporter construct used in **e**–**g**. Impaired U7 snRNA transcript cleavage by INT at the 3′-box leads to increased Renilla Luciferase (RLuc) levels. PAS polyadenylation signal. **e**, **f** Luciferase assay using the U7-reporter in cells depleted for individual INT subunits **e** or two INTS10–INTS13–INTS14 module subunits **f**. A plasmid expressing Firefly luciferase (FLuc) under control of a CMV promoter was used as transfection control. Statistical significance was determined for each knockdown relative to the control. **g** Rescue assays for INTS13 function in snRNA processing using the U7-reporter. Cells were treated with a control siRNA or INTS13 siRNA and transfected with HA-tagged INTS13 wt or mutants together with the reporter and FLuc transfection control. MBP served as negative control. All bar graphs depict mean values of biological triplicates shown together with individual values as dots, and error bars representing standard deviations. Statistical significance was determined by unpaired, two-tailed *t*-test: not significant (ns) *p* ≥ 0.05; significant *p ≤ 0.05, **p ≤ 0.01, ***p ≤ 0.001, ****p ≤ 0.0001. Western blots for all experiments and immunofluorescence assays for INTS13 mutants are shown in Supplementary Fig. 8. Source data are provided as a Source Data file.

After establishing that INTS10–INTS13–INTS14 form a stable module in nuclei, we wanted to test whether they also behave like a functional unit. We first aimed at characterizing effects of individual protein knockdowns (Fig. 6a, Supplementary Fig. 8a, b) on UsnRNA cleavage. Mature and misprocessed, i.e. 3′-extended forms of U1 snRNA were detected by RT-qPCR using specific primer pairs (Fig. 6b, c), similar to described assays^25^. INTS13 and INTS14 depletions led to mutual downregulation consistent with their tightly entwined structure but all other depletions are independent of one another (Fig. 6a, Supplementary Fig. 8a, b). As expected based on previous reports^9,35,45^, depletion of the endonuclease INTS11 leads to a pronounced increase of U1 misprocessing by more than 15-fold compared to the control siRNA (Fig. 6c). Knockdown of INTS7 as one of the INT core subunits has a smaller effect on misprocessing (5.5-fold increase). In comparison, effects of INTS10, INTS13, or INTS14 knockdown on U1 3′-end formation are more modest, increasing abundance by 2.2, 3.2, and 3.7-fold, respectively. This observation is consistent with published observations for some of these subunits^8,9,27,35,45^ and indicates that they have a more accessory role during INT-mediated cleavage of UsnRNAs.

To facilitate functional testing of the module, we also employed a more sensitive reporter assay that yields a stable transcript when processing by INT is impeded, analogous to published approaches^6,9,45^. The reporter consists of the U7 snRNA under control of its endogenous promoter followed by the 3′-box processing signal and a Renilla luciferase (RLuc) gene with a strong poly-adenylation signal (Fig. 6d). RLuc expression from this reporter is inversely dependent on INT cleavage efficiency. To validate the reporter, we repeated the individual depletions of INTS and obtained a qualitatively similar picture of their differential contributions on UsnRNA processing (Fig. 6e, Supplementary Fig. 8b). Again INTS11 depletion has the strongest effect (278-fold increase in RLuc signal), while INTS7 knockdown is weaker (70-fold) and INTS10–INTS13–INTS14 depletions yield modest effects (3.8, 3.3, and 4.3-fold). For further validation of the reporter plasmid, removal of the PSE resulted in a strong reduction of the RLuc signal, demonstrating that U7 and RLuc expression are driven by the U7 promoter on the plasmid (Supplementary Fig. 7d–f). In contrast, deletion of the 3′-box increases RLuc levels and makes RLuc expression less responsive to INTS11 depletion, demonstrating that U7 transcript termination depends on INT-mediated cleavage.

Using the U7 reporter, we next performed codepletions to test whether INTS10, INTS13, and INTS14 act together (Fig. 6f and Supplementary Fig. 7c). INTS10 codepletion with either INTS13 or INTS14 leads to an approximately additive increase of RLuc levels, suggesting that INTS10–INTS13–INTS14 form a functional module in the context of the INT holo-complex but have additive roles.

### INTS13 binds the cleavage module during UsnRNA processing

In a previous study, INTS13 was shown to be required for embryonic development in flies and frogs probably via impairing cell cycle progression^36^. Engineered mutant flies, which lack only the last 64 residues of the INTS13 C-terminus, develop into adults and are viable but defective in spermatogenesis^37^. Interestingly, these deleted C-terminal residues correspond exactly to the CMBM that we identified here, suggesting that the role of INTS13 during meiosis is dependent on its interaction with the INT cleavage module. Therefore, we wanted to test whether its function in UsnRNA processing would also be hampered by deleting the CMBM. We used the U7 reporter for an INTS13 rescue assay (Fig. 6g and Supplementary Fig. 8h, i). Depletion of INTS13 increased RLuc expression more than two-fold, which was almost completely rescued by cotransfection of INTS13 wt, while an INTS13 mutant defective for INTS14 binding (INTS13 P2+P4) showed a slightly reduced rescue efficiency. In contrast, the INTS13 ΔCMBM mutant that cannot bind the cleavage module is not able to rescue UsnRNA cleavage at all, even though it is expressed at similar levels as wt (Supplementary Fig. 8h) and correctly localizes to the nucleus (Supplementary Fig. 8i).

Taken together with previous data, our observations suggest that INTS13′s most important function is to bind the cleavage module. Although the INTS10–INTS13–INTS14 module is not as central to UsnRNA processing as the catalytic subunit INTS11, its depletion impairs INT in this process and improper INT assembly due to disruption of the connection between INTS13 and the cleavage module decreases the efficiency of UsnRNA maturation.

### INTS10–13–14 are important for termination after pausing

**Fig. 7:**
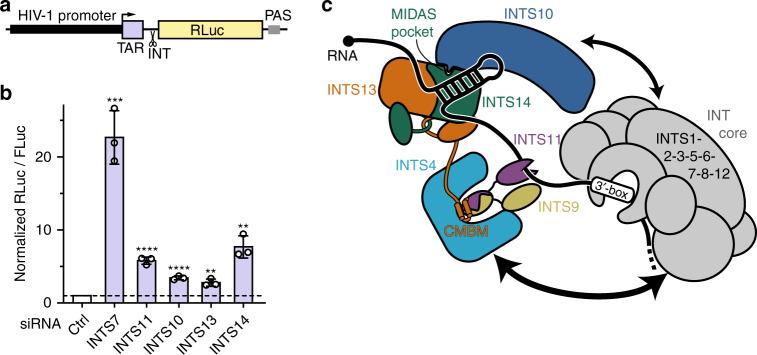
INTS10–13–14 are required for termination after pausing. **a** Scheme of the RNAPII pause-termination reporter used in **b**. Reduced RNAPII pausing and termination at the HIV-1 TAR element results in elongation and increased RLuc levels. **b** Luciferase assay using the TAR pause-termination reporter. Cells depleted of individual INT subunits were transfected with the TAR reporter and FLuc transfection control plasmid. Statistical significance was determined for each knockdown condition relative to control. Bars show mean values of biological triplicates together with the individual replicates as dots. Error bars represent standard deviations. Statistical significance was determined by unpaired, two-tailed *t*-test: not significant (ns) *p* ≥ 0.05; significant *p ≤ 0.05, **p ≤ 0.01, ***p ≤ 0.001, ****p ≤ 0.0001. Western blots in Fig. 6a. Source data are provided as a Source Data file. **c** Summary model of INT modules and their respective protein interaction networks. INTS10–INTS13–INTS14 brings cleavage module and target RNA into proximity.

A second function of INT, in addition to UsnRNA processing, is the termination of RNAPII transcription after promoter-proximal pausing on protein-coding genes^9,10,13^. In order to test whether the INTS10–INTS13–INTS14 complex plays a role in this process, we made use of the well-characterized HIV-1 transactivation-response (TAR) stem loop, which induces RNAPII pausing via direct recruitment of negative elongation factor (NELF) subunit E^46,47^. We generated a reporter plasmid, in which the HIV-1 promoter and TAR element are placed in front of an RLuc gene followed by a PAS (Fig. 7a), analogous to published reporters^13,48^. As a control, expression of HA-tagged HIV-1 Tat releases RNAPII from pausing at TAR into processive elongation^47^, resulting in a strong (23.6-fold) increase of RLuc levels (Supplementary Fig. 8j, k). Knockdown of the core component INTS7 led roughly to the same increase of RLuc levels as the coexpression of HIV-1 Tat (Figs. 6a and 7b), indicating that INT is a major factor that prevents RNAPII pause release from the TAR element. Interestingly, depletion of the endonuclease subunit INTS11 has a milder effect on RLuc expression (six-fold), suggesting that although transcript cleavage plays an important role for the process, it is not the main event that triggers transcription termination after pausing. Similarly, INTS11 was observed to have a lower contribution to transcription regulation than core INTS for several mRNA transcripts in *Drosophila*^9^. Knockdown of INTS10, INTS13, or INTS14 results also in lower RLuc signals than INTS7 (3.7-fold, 3.4-fold, and 9.4-fold increase, respectively). However, INTS14 depletion increases RLuc levels more than the catalytic subunit INTS11. This observation suggests, that the module has more functions during RNAPII pause termination in addition to binding of the INT nuclease module, and these remain to be identified in the future.

## Discussion

In this study, we identified a new module of INT that is composed of INTS10–INTS13–INTS14 and revealed its function as a DNA/RNA-binding module. We characterized its molecular architecture in detail and defined its connection to other INT components. Together with literature data, we can now draw a significantly more detailed picture of INT’s molecular organization and function (Fig. 7c):

Firstly, INTS13 and INTS14 form a tightly entwined and physically interlinked heterodimer that binds INTS10 via the MIDAS pocket of the INTS14 VWA domain. This observation has implications for previously suggested INTS13 functions outside of INT during cell differentiation^35^. The interdependent folding of the INTS13–INTS14 complex, together with the chain interlink, indicate that INTS13 will most likely not exist outside of a complex minimally with INTS14. In support of this notion, we found that both proteins strongly stabilize each other in human cells. Furthermore, INTS14 shows the strongest enrichment in IP-MS of INTS13, both in differentiated and undifferentiated cells^35^. INTS10 is the second most strongly enriched protein in these IP-MS datasets. Given that we observe a strong and stable complex between INTS13–INTS14 and INTS10 both in vitro and in nuclear extracts, and that codepletions affect UsnRNA processing additively, we propose that INTS10–INTS13–INTS14 form one stable functional unit in cells.

Secondly, INTS13 is responsible for connecting the subcomplex to the cleavage module through its C-terminal CMBM and this section of INTS13 is necessary for efficient UsnRNA 3′-end processing. As mentioned above, deletion of exactly this region in flies disrupts spermatogenesis and leads to sterile offspring^37^. In these mutant flies and also in INTS13-depleted human cells, dynein–dynactin recruitment to nuclear envelopes is aberrant during cell division^37,38^. It was further shown that this dynein-misrecruitment phenotype is mirrored by depletion of other INTS in human cells, suggesting that INTS13 is required for faithful cell division within the context of the holo–INT complex^39^. Our analysis now suggests that the lack of INT cleavage module binding by INTS13 is the molecular basis for its impaired function that compromises meiosis and mitosis. Given the broad roles that INT plays during RNAPII transcription of ncRNAs and mRNAs^2,9,10^, it is possible that the resulting phenotypes are then outcomes of transcript misregulation, due to defective holo–INT assembly on RNAPII during transcription of specific genes.

Our data also holds the possibility that nuclease module interaction with INTS13 could be involved in INTS13′s function in myeloid differentiation in human cells^35^. Consistent with this notion, in the respective study INTS11 and several INT core subunits were detected in an IP of co-activator NAB2, which mediates INTS13 binding to monocytic enhancers during cell commitment. In part INTS13 was suggested to act independent of INTS11 in the same study, because no interaction between the endonuclease module and INTS13 could be detected in IPs from fractionated nuclear extract^35^. However, our data could potentially explain this observation since the INTS13 antibody used in these IPs specifically recognizes the CMBM and thus could possibly interfere with cleavage module binding (see also Western blot using the same antibody, Supplementary Fig. 7h, lane 5). Nevertheless, INTS13 ChIP sites at enhancers only partially overlapped with INTS11 and thus an additional cleavage-module-independent function cannot be ruled out and requires further investigation.

Thirdly, the INTS10–INTS13–INTS14 module binds to DNA and RNA, preferentially to RNA stem loop regions, possibly stabilizing association of the cleavage module with target RNAs. This notion is consistent with earlier literature on UsnRNA-processing requirements, which demonstrated that terminal stem loops within U2 and U7 snRNAs promote 3′-end processing^45,49^. Similarly, pre-miR-HSUR4 cleavage was shown to depend on proper distancing between the pre-miRNA stem loop and the 3′-box-like cleavage signal^50^. Our data could suggest that the INTS10–INTS13–INTS14 module’s binding preference might be the molecular basis behind increased INT processing efficiency after a hairpin structure.

In summary, our work revealed a separable and stable INT module, characterized it structurally and biochemically, and identified its physical connection to the RNA cleavage module of INT, thereby providing molecular insight for a mechanistic understanding of INT function during RNAPII transcription.

## Methods

### Antibodies

The following antibodies and dilutions were used for western blotting: horseradish peroxidase-linked anti-HA antibody (Roche, 12013819001, dilution 1:5000), rabbit anti-INTS1 (Bethyl, A300-361A, dilution 1:2000), rabbit anti-INTS4 (Bethyl Laboratories, A301-269A, dilution 1:2000), rabbit anti-INTS7 (Bethyl, A300-271A, 1:1000), rabbit anti-INTS9 (Proteintech, 11657-1-AP, dilution 1:2500), rabbit anti-INTS10 (Proteintech, 15271-1-AP, dilution 1:2000), rabbit anti-INTS11 (Bethyl, A301-274A, dilution 1:1000), rabbit anti-INTS13 (Bethyl, A303-575A, dilution 1:5000), rabbit anti-INTS14 (Bethyl, A303-576A, dilution 1:2500), goat anti-GST (GE Healthcare, 27-4577-01, dilution 1:1000), mouse anti-V5 (AbD Serotec, MCA 1360, dilution 1:1000), mouse anti-β-actin (Proteintech, 60008-1-Ig, dilution 1:20,000). Secondary antibodies: horseradish peroxidase-linked anti-mouse IgG (Sigma, A9044, dilution 1:5000), anti-rabbit IgG (Sigma, A9169-2ML, dilution 1:5000) or anti-goat IgG (Sigma, A8919, dilution 1:5000) antibodies were used. For immunofluorescence the following dilutions and antibodies were employed: rat anti-HA (Roche, 11867423001, dilution 1:200), rabbit anti-INTS13 (Bethyl, A303-575A, dilution 1:250), mouse anti-Dynein IC (Millipore, MAB1618, dilution 1:500), Alexa Fluor 488-labeled goat anti-mouse (Invitrogen, A11001, dilution 1:300), Alexa Fluor 568-labeled goat anti-rabbit (Invitrogen, A11011, dilution 1:300), and Alexa Fluor 633-labeled goat anti-rat (Invitrogen, A21094, dilution 1:300).

### DNA constructs

Genes encoding full-length human INTS4, INTS9, INTS11, INTS13, and INTS14 were cloned by reverse transcription from total RNA of HeLa cells using gene-specific primers. The INTS10 gene was acquired from the human open-reading frame library (hORFeome V5.1, ID: 3858). Truncated constructs were PCR amplified from the full length genes using gene-specific primers. Construct boundaries were the following: INTS14 VWA (1–210), INTS14 ΔVWA (211–518), INTS13 C-term (566–706), INTS13 CMBM (649–694), and INTS13 ΔCMBM (1–648). All generated constructs were confirmed by sequencing.

To express INTS4, INTS9, INTS10, INTS11, INTS13, and INTS14 in human cells, the respective full-length cDNAs or truncations were inserted into *Xho*I and *Not*I sites of the pCIneo-λN-HA or pCIneo-V5-SBP plasmids (kind gifts from Elisa Izaurralde^51,52^), resulting in fusion proteins with N-terminal λN HA or V5-SBP tags. Mutations in INTS13 and INTS14 were introduced by the QuikChange mutagenesis PCR method (Agilent) using appropriate primers.

For recombinant protein expression in insect cells, INTS4, INTS9, INTS11, INTS10, INTS13, and INTS14 were cloned and assembled in multigene expression cassettes by ligation-independent cloning (LIC) using the MacroBac system^53^. Genes of interest were PCR amplified introducing LIC-compatible overhangs. Modified and *Ssp*I-linearized pFastBac vectors (empty or containing N-terminal tandem Strep tag (2S) or deca-His tag (His_10_) that are cleavable by HRV 3C protease) served as a destination vectors. Inserts and vectors were treated with T4 DNA polymerase, annealed and subsequently transformed into *E. coli* DH5α. For larger gene assemblies, expression cassettes were consecutively added by restriction digest followed by LIC cloning. Expression vectors containing 2S-INTS13–INTS14, INTS10–(2S)-INTS13–INTS14, and His_10_-INTS4–INTS9–(2S)-INTS11 were assembled. Bacmids were obtained after transformation of the expression vectors into DH10Bac cells^54^ and blue-white colony screening. Correct insertion of expression cassettes into bacmids was verified by PCR.

For the expression of recombinant proteins in *E. coli*, cDNA encoding INTS10 was inserted between *Nhe*I and *Nde*I restriction sites of pnEA-pM^55^, and cDNA encoding INTS13 (649–694) or INTS14 (1–210) truncations were inserted between *Xho*I and *BamH*I restriction sites of pnEA-pG^55^, resulting in fusion proteins containing N-terminal MBP and GST tags, respectively, that are cleavable by the HRV 3C protease.

For in vitro RNA transcription, the U1-stem loop 4 (SL4), 3′-box or the HSUR4 miRNA precursor (pre-miR)^7^ were cloned by inserting annealed DNA oligos (Sigma) that encoded the T7 promoter and the target RNA into *EcoR*I and *Nco*I sites of pSP64-T7HDV (kind gift of Oliver Weichenrieder^56^). Primer sequences used were TAATACGACTCACTATAGGGTGGGGGACTGCGTTCGCGCTTTCCCCTGGCCGG (U1 SL4), TAATACGACTCACTATAGGACTTTCTGGAGTTTCAAAAACAGACTGTACGCCAGCCGG (U1 3′-box), and TAATACGACTCACTATAGGCGTGTTGCTACAGCTATAAACTTCAAACATGCAGTTTATAGCAGTGGGCAACACGT (pre-miR-HSUR4).

The U7 snRNA luciferase reporter construct was generated from the psiCheck-2 vector (Promega). After removal of the firefly luciferase gene including its promoter, the SV40 promoter and chimeric intron in front of the RLuc open-reading frame were replaced by the human U7 snRNA preceded by its upstream promoter region (500 nt) and followed by its downstream region including the 3′-box (42 nt). Start codons after the U7 snRNA were mutated apart from one within the 3′-box motif, which was placed in frame with the downstream RLuc. Deletions of the PSE (nt −61 to −42 from the TSS) and 3′-box (nt +72 to +90 from the TSS) were generated by mutagenesis PCR using primers GGAACAAGAAAAAAGTCACCTAAGAGTTCCTTTATATCCCATCTTCTC, GGAACAAGAAAAAAGTCACCTAAGAGTTCCTTTATATCCCATCTTCTC and CGGAAAGCCCCTCTTATGATTTGTTTTCACTGTGCCATATGAAAC, GTTTCATATGGCACAGTGAAAACAAATCATAAGAGGGGCTTTCCG, respectively.

For the HIV-LTR reporter construct, the U7 promoter, snRNA, and 3′-box upstream of RLuc were replaced with the HIV-1 promoter and TAR element. Promoter (636 nt) and TAR (120 nt) were amplified from plasmid HIV-1 LTR-gfp (Addgene #115809)^57^ using primers AAAAAAGGATCCACCTAGAAAAACATGGAGCAATCAC and AAAAAAGCTAGCCAACAGACGGGCACACACTAC and cloned into *Bgl*II and *Nhe*I restriction sites of the reporter plasmid. The HIV-1 Tat protein was also amplified from plasmid HIV-1 LTR-gfp and its C-terminus extended to the full length sequence using overlapping PCR primers. It was then cloned into *Xho*I/*Not*I restriction sites of pCIneo-λN-HA.

### Large scale protein expression and purification

Protein complexes (2S-INTS13–INTS14, INTS10–2S-INTS13–INTS14 or His_10_-INTS4–INTS9–2S-INTS11) for structure determination and interaction studies were expressed in insect cells. Bacmid DNA isolated from *E. coli* DH10Bac cells was used to transfect Sf9 cells (ThermoFisher) using EscortIV transfection reagent (Merck) growing in SF-4 Baculo Express ICM medium (BioConcept) to generate baculovirus. Protein expression was carried out in HighFive cells (ThermoFisher) at 130 rpm and 27 °C for 48 h post-infection. Cells were harvested by centrifugation, and resuspended in lysis buffer (50 mM HEPES pH 7.5, 200 mM NaCl, 2 mM DTT) supplemented with 1xEDTA-free protease inhibitor (Merck) and 5 μg/mL DNase I (Roche). After lysis by sonication, the crude lysate was cleared by centrifugation and filtered (0.45 µm). Proteins were bound to a pre-equilibrated StrepTrap column (GE Healthcare), washed with lysis buffer, and the respective protein complexes were eluted in lysis buffer containing 2.5 mM *d*-desthiobiotin. If needed, protein tags were removed by overnight cleavage with HRV 3C protease. The protein complex was diluted into heparin buffer (50 mM HEPES pH 7.5, 100 mM NaCl, 2 mM DTT) and was subsequently purified over a heparin column (GE Healthcare). Complexes were eluted by a linear gradient to 1 M NaCl and subjected to a final gel-filtration step (Superose 6, GE Healthcare) in gel-filtration buffer (10 mM HEPES pH 7.5, 200 mM NaCl, 2 mM DTT). The complex was either used directly to set up crystallization plates, in biochemical assays or flash-frozen in liquid nitrogen and stored at −80 °C.

### Copurification assays

For interaction studies, MBP-INTS10, GST-INTS13 (649–694), or GST-INTS14 (1–210) constructs were expressed separately in BL21 Star (DE3) cells (Invitrogen) harboring the corresponding plasmids. Cells were grown at 37 °C in LB medium until an OD_600_ of 0.3 was reached. Protein expression was induced with 2 mM isopropyl-β-d-thiogalactopyranosid (IPTG) and continued overnight at 20 °C. 2S-INTS4, 2S-INTS9, 2S-INTS11, 2S-INTS9–(2S)-INTS11, His_10_-INTS4–INTS9–(2S)-INTS11, 2S-INTS13–INTS14 or INTS10–(2S)-INTS13–INTS14 were expressed in insect cells as described above. Cell pellets were resuspended in lysis buffer and lysed by sonication as described above. The cleared lysates or purified components were mixed with 50 µL glutathione sepharose 4B resin (50% slurry, Amersham Biosciences) or strep-tactin sepharose (50% slurry, IBA Lifesciences). Purified GST or Strep-MBP served as negative controls. Proteins and beads were incubated for 30 min on ice before they were washed four times with 700 µL lysis buffer. Bound complexes were eluted in lysis buffer containing 25 mM glutathione and 2 mM biotin, respectively, and precipitated with trichloroacetic acid. Precipitates were resuspended with protein sample buffer before separation by SDS–PAGE and detection by Coomassie staining or Western blotting.

### In vitro transcription of RNAs

Template plasmids were linearized with *Nco*I (to solely transcribe the short RNAs) and purified with a PCR clean-up kit (Macherey-Nagel). RNAs were in vitro transcribed by T7 run-off transcription for 2 h at 37 °C. Reactions contained 80 µg DNA template, 56 µg T7 RNA polymerase (home made), 21 mM MgCl_2_, 3.5 mM of each NTP, 40 mM Tris–HCl pH 8.0, 1 mM spermidine 0.01% Triton-X, and 5 mM DTT. RNA products were extracted with phenol–chloroform, ethanol precipitated and resuspended in water. Purity of the RNA was checked by inspecting the UV spectrum and on a denaturing acrylamide gel.

### Electrophoretic mobility shift assay

U1 SL4 and pre-miR-HSUR4 were refolded in 10 mM HEPES pH 7.5, 1 mM EDTA by incubation at 75 °C for 5 min and cooling down to 20 °C at a rate of 0.5 °C/10 s. Complementary DNA oligos were annealed to obtain dsDNA in 10 mM HEPES pH 7.5, 100 mM NaCl, 2 mM MgCl_2_ using the same procedure. Complete annealing and folding was checked on a native acrylamide gel. For binding, in vitro transcribed RNA or DNA oligos (Sigma, U1 3′-box ACTTTCTGGAGTTTCAAAAACAGACTGTACGCCA) were diluted with assay buffer (50 mM HEPES pH 7.5, 200 mM NaCl, 2 mM MgCl_2_). For each NA tested, a series of reactions were prepared on ice, each containing 2 µM RNA or 1 µM DNA, 0.5 μL 10× loading dye (0.4% (w/v) orange G, 50% (v/v) glycerol, 1 mM EDTA) and 3.5 μL of serially diluted protein. Samples were incubated on ice for 30 min prior to analysis by native 4–12% TBE PAGE (Invitrogen, 20 min, 150 V). Gels were then incubated in 1xSYBR Gold in TE buffer and were subsequently scanned with a Typhoon FLA-7000 (GE Healthcare).

### Fluorescence anisotropy assays

Binding reactions were carried out with 10 nM 5′-6-FAM-labeled U1 3′-box RNA (CUGGAGUUUCAAAAACAGACUG) or U_12_ RNA (Microsynth) in binding buffer (10 mM HEPES pH 7.5, 5% glycerol, 2 mM MgCl_2_) containing 50 mM NaCl or no additional salt. Proteins at concentrations ranging from 10 nM to 30 μM were briefly incubated with the RNA in a black 384-well plate (Greiner) in a total reaction volume of 30 μL. FP was determined with a CLARIOstar microplate reader (BMG Labtech, Software version 5.40-1) by excitation at 482 nm and detection at 530 nm. Measurements on the same sample were repeated up to five times and all samples were prepared in triplicate. After baseline substraction, FP values were normalized to 1 using Microsoft Excel (2016). Mean values of experimental triplicates and their standard deviation were plotted against the protein concentration and fitted using GraphPad Prism (Version 8.4.2) to a Hill equation^58^:

1 $${\rm{FP}} = \frac{{\left( {\frac{{\left[ {\rm{protein}} \right]}}{{K_{\mathrm{{d}}}}}} \right)^{\rm{{h}}}}}{{1 + \left( {\frac{{\left[ {\rm{protein}} \right]}}{{K_{\mathrm{{d}}}}}} \right)^{\rm{{h}}}}}$$

### Protein XL–MS

INTS13–INTS14 and INTS4–INTS9–INTS11–INTS13 CMBM complexes were diluted in 10 mM HEPES pH 7.5, 200 mM NaCl, 2 mM DTT to ~1.0 mg/mL total protein concentration for cross-linking. Experiments were carried out at the 50–75 μg scale. Cross-linking with DSS (*d*_0_/*d*_12_, creative molecules) was carried out at a final concentration of 1 mM DSS for 30 min at 37 °C^59^. The reaction was stopped by addition of ammonium bicarbonate to 50 mM final concentration and additional incubation for 30 min at 37 °C. Cross-linked samples were evaporated to dryness in a vacuum centrifuge before reconstitution in 8 M urea. Disulfide bonds were reduced by tris(2-carboxyethyl)phosphine hydrochloride and free thiol groups alkylated with iodoacetamide. Reduced and alkylated proteins were digested with different proteases as follows:

*Trypsin/Lys-C*: After dilution to 5.5 M urea with 150 mM ammonium bicarbonate, endoprotease Lys-C (Wako) was added at an enzyme-to-substrate ratio of 1:100 and the sample was incubated for 2.5 h at 37 °C. The solution was further diluted to 1 M urea with 50 mM ammonium bicarbonate, trypsin (Promega) was added at an enzyme-to-substrate ratio of 1:50 and the sample was incubated overnight at 37 °C.

*Asp-N (for INTS13-INTS14 only)*: After dilution to 1 M urea with 50 mM ammonium bicarbonate, endoprotease Asp-N (Promega) was added at an enzyme-to-substrate ratio of 1:50 and the sample was incubated overnight at 37 °C.

After the digestion, samples were acidified with 2% formic acid (v/v) and purified by solid-phase extraction (Sep-Pak tC18, Waters). Samples were then fractionated by size-exclusion chromatography on a Superdex Peptide PC 3.2/30 column (GE), as described previously^59,60^. Three fractions were collected for liquid chromatography–tandem MS (LC–MS/MS) analysis. Despite extensive tests with a number of different proteases and additional crosslinking of acidic surface residues^61^, the INTS14 VWA is largely inaccessible to the XL–MS approach, due to the low number of cross-linkable surface residues and long peptides obtained upon protease digestion.

All LC–MS/MS analyses were carried out on an Orbitrap Fusion Lumos mass spectrometer with Xcalibur version 4.2.28.14 and an Easy-nLC 1200 HPLC system (both ThermoFisher Scientific). The stationary phase was an Acclaim PepMap RSLC C18 column (250 mm × 75 μm, ThermoFisher Scientific) and the mobile phases were *A* = water/acetonitrile/formic acid (98:2:0.15, v/v/v) and *B* = acetonitrile/water/formic acid (80:20:0.15, v/v/v). Gradient elution was performed by adjusting the percentage of *B* from 11% to 40% in 60 min. The flow rate was set to 300 nl/min.

MS data was acquired in the data-dependent acquisition mode with the following parameters: MS data was acquired in the Orbitrap analyzer at a resolution of 120,000. Precursors with a charge state of +3 to +7 were dynamically selected for fragmentation in top speed mode with a 3 s cycle time. Precursors were isolated in the selection quadrupole with an isolation width of 2.0*m*/*z* and fragmented in the linear ion trap at 35% normalized collision energy. Fragment ions were detected in the linear ion trap in rapid resolution mode. Dynamic exclusion was activated for 30 s after one scan event. For the INTS4–INTS9–INTS11–INTS13CMBM complex, one replicate was acquired with high-resolution fragment ion detection in the Orbitrap analyzer at a resolution of 30,000.

MS/MS spectra were searched against a sample-specific protein database containing the two INTS and contaminant proteins (4 human keratins, 1 insect protein) using the dedicated search engine, xQuest^62^. Contaminant proteins were identified from a standard database search against the UniProt/SwissProt database with Mascot (version 2.5.1, MatrixScience), and identifications with a Mascot score of 200 or higher were included. Contaminant protein sequences were retrieved from UniProt while the actual sequences of the constructs were used for the INTS. A decoy database was generated by first reversing and then shuffling the protein sequences using the xdecoy.pl script from xQuest.

xQuest was configured with DSS−*d*_0_/*d*_12_ as the cross-linker, with the respective mass shifts for cross-linked and dead-end products and the mass differences between light and heavy forms of the linkers. DSS was specified to react with Lys residues and the proteins’ N-termini. Protease cleavage rules were defined as follows: Lys-C/trypsin—cleavage C-terminal to Lys and Arg, except if followed by Pro; Asp-N—cleavage N-terminal to Asp and Glu. The number of allowed missed cleavages (per peptide) was set to 2 for Lys-C/trypsin, and 4 for Asp-N. The initial allowed MS mass tolerance was 15 ppm and the MS/MS tolerance was set to 0.2 Da for common ions and 0.3 Da for cross-link ions for fragment ion detection in the ion trap and 20 ppm for fragment ion detection in the Orbitrap.

After the initial search, a filter of TIC ≥ 0.1 was applied. The MS mass tolerance window was reduced to ±5 ppm or less based on the experimentally observed distribution. Spectra of all candidate assignments (including decoy hits) were manually evaluated. Identifications with ≥4 bond cleavages per peptides overall, or ≥3 consecutive bond cleavages, were kept. False discovery rates (FDR) were determined based on validated target and decoy hits and adjusted to ~5%. The FDR estimate is affected by the small number of decoy hits and the composition of the target database, because most decoy hits involve contaminant proteins.

### Crystallization and structure determination

Initial screens were carried out using the sitting drop vapor diffusion method using 11.1 mg/mL of the 2S-INTS13–INTS14 complex. The 200 nL protein was added to 200 nL of reservoir solution. Morphologically similar crystals appeared within one day in several different conditions. The best diffracting crystals were optimized in 0.1 M Bis–Tris (pH 6.0), 0.75 M (NH_4_)_2_SO_4_, and 1% (w/v) PEG3350. Crystals were cryoprotected using reservoir solution supplemented with 15% (v/v) glycerol and flash-frozen in liquid nitrogen.

Diffraction data were recorded on a PILATUS 6M detector and DA+ software at the PXIII beamline of the Swiss Light Source (SLS) at a temperature of 100 K. Data were processed using XDS and XSCALE (both Version January 26, 2018)^63^. Initial phasing was achieved using multiwavelength anomalous dispersion from data recorded on three isomorphous crystals, one native, plus one Au and one Ta derivative (Supplementary Table 1). SHELXD (Version 2013/2)^64^ was used to locate gold and tantalum sites. The sites of the two derivatives were placed on the same origin by inspecting the initial maps and transposing the gold sites correspondingly. Phases of the two derivatives were combined using MLPHARE in CCP4 (Version 7.0)^65^. The resulting map was used to build several visible secondary structure elements and place homology models of two VWA domains in COOT (Version 0.8.9.2)^66^. In parallel, the combined phases were used to calculate an anomalous difference density map from a sulfur-SAD dataset that was generated by merging highly redundant datasets from two isomorphous crystals (Supplementary Table 1). This difference density map was used to identify chains and assign sequences to identifiable helices based on their methionine/cysteine content. The rudimentary model was subsequently used to locate all sulfur sites with PHASER EP (Version 2.8.2)^67^ using the sulfur-SAD dataset and calculate improved phases. The resulting map was of sufficient quality to build the remainder of the structure. Correct sequence assignment was ensured using sulfur sites in the anomalous difference density map as markers. Strong peaks in the anomalous density on the protein surface were used to place six sulfate ions from the crystallization condition in the density. Iterative cycles of model building in COOT and refinement performed with Phenix (Version 1.15.2-3472)^68^ against the high-resolution native dataset were then used to finalize the structure. The final model contains residues 1–564 of INTS13 and 2–512 of INTS14, with the exception of a few disordered surface loops that were omitted from the model (INTS13 residues 34–40, 268–278, 295–311, 366–369, 515–522, and INTS14 residues 288–296). Due to the sparse crystal packing and high solvent content of the crystal (78%, Supplementary Fig. 1b) overall *B*-factors are comparably high (mean *B* = 81.6). Consequently, several side chains in surface loops with poor densities (~3.5%) were modeled as stubs.

### Coprecipitation assays

All human cells were cultured in Dulbecco’s modified Eagle’s medium (DMEM, Sigma) supplemented with 10% fetal calf serum, 100 U/mL penicillin, and 100 mg/mL streptomycin (Thermo Scientific). For coprecipitation assays, 2.7 × 10^6^ HEK293T cells were seeded in 10 cm dishes and transfected 24 h post-seeding using the calcium phosphate method. To express V5-SBP and λN-HA-tagged proteins, cells were transfected with 20 μg of total plasmid. V5-SBP-tagged maltose-binding protein was used as a negative control. Two days after transfection, cells were lysed for 10 min on ice in RIPA buffer (50 mM HEPES pH 7.6, 150 mM NaCl, 1% NP-40, 0.5% sodium deoxycholate, 2 mM DTT) if overexpressed proteins were detected or in NET buffer (50 mM Tris–HCl pH 7.5, 150 mM NaCl, 0.1% Triton X-100, 10% glycerol, 2 mM DTT) if endogenous proteins were detected. For lysis both buffers were supplemented with 1x EDTA-free protease inhibitor (Merck), 5 μg/mL DNase I and 200 μg/mL RNaseA (Qiagen). After mild sonication, cell lysates were centrifuged at 16,000 × *g* for 15 min at 4 °C. The cleared lysate was rotated for 1 h at 4 °C in the presence of 50 μL of streptavidin sepharose beads (50% slurry, GE Healthcare). Beads were washed three times with RIPA or NET buffer, respectively. Bound proteins were eluted with 100 μL of protein sample buffer. For further analysis, proteins were separated by SDS–PAGE and detected by Western bloting.

### Fractionation of nuclear extract

Nuclear extract from 2 mL of HEK293T cell pellet was essentially prepared as described^69^, with the difference that after sucrose cushioning the nuclei were directly taken up in 4 mL fractionation buffer (50 mM HEPES–KOH pH 7.9, 500 mM KCl, 1.5 mM MgCl_2_, 0.2 mM EDTA, 10% (v/v) glycerol, 0.5 mM DTT) supplemented with 5 μg/mL DNaseI. To disrupt the released chromatin, the suspension was sonicated 3 × 30 s on ice and centrifuged 10 min at 16,000 × *g* and 4 °C. Subsequently, 0.4 mL of HEK293T nuclear extract (2.5 mg/mL protein) were loaded onto a Superose 6 10/300 GL column (GE Life Science) pre-equilibrated in fractionation buffer. Flow rate was fixed at 0.3 mL/min, and 0.4 mL fractions were collected. Two identical runs were performed and equivalent fractions of both runs were pooled, TCA precipitated and all even fractions were analyzed by SDS–PAGE and Western blotting.

### RT-qPCR and luciferase reporter assays

For single protein depletion assays, 3.5 × 10^5^ HeLa cells were seeded per well of a six-well plate in 2 mL DMEM one day before transfection with 20 nM siRNA using Lipofectamine RNAiMAX (Invitrogen) according to manufacturer’s instructions. The following siRNAs (Microsynth, Eurofins) were used to deplete target proteins: INTS7 GGCUAAAUAGUUUGAAGGA^34^, INTS11 GAAAUGGGCCGGAAACGAA, INTS10 GGAUACUUGGCUUUGGUUA^34^, INTS13 CAGCAAGAUGGUAUAGUUA targeting the 3′-UTR^39^, and INTS14 GGCAGAUUUUUACUAUUGA. The MISSION Universal Negative Control #1 siRNA (Sigma) served as control. One day after transfection cells were detached with trypsin/EDTA (Invitrogen) and reseeded into new plates at 6 × 10^5^ cells per well. The following day cells were transfected with 20 nM (ctrl, INTS11, INTS10, INTS14) or 40 nM siRNA (INTS13) using Lipofectamine RNAiMAX. Cells were harvested one day later by either lysing them directly in protein sample buffer for Western blot analysis or with TRIzol (Invitrogen) for RNA extraction. Total RNA was extracted using phenol–chloroform, DNaseI treated and purified. After reverse transcription using random hexamer primers (Thermo Scientific) and Affinity Script RT (Agilent), mature and misprocessed U1 snRNA were detected by qPCR using KAPA SYBR FAST (Roche) in a CFX96 real-time PCR machine (Bio-Rad) in technical triplicates. qPCRs were carried out on three biological replicates and from each of these the mean of the respective technical replicates was used to calculate the mean, standard deviation, and statistical significance for the biological triplicate. For all three replicates using Microsoft Excel, ΔΔC_T_ values were determined versus mature U6 snRNA, which as an RNA polymerase III transcript is not targeted by INT. qPCR primers (Sigma) were ATACCATGATCACGAAGGTGGTT, CAGTCCCCCACTACCACAAATTA (U1)^25^, TACCTGGCAGGGGAGATACC, GCGTACGGTCTGTTTTTGAAACTC (U1mis)^25^, and AATATGGAACGCTTCACGAAT, ATTGGAACGATACAGAGAAGATTA (U6)^70^.

For luciferase assays, the second transfection included 0.4 µg U7-RLuc or HIV1-TAR reporter plasmids and 0.2 µg pEGFP-N3-FLuc (transfection control, CMV driven FLuc, kind gift from Elisa Izaurralde^71^) and Lipofectamine 2000 (Invitrogen) was used according to manufacturer’s instructions. For harvest, cells were lysed in 0.2 mL 1x passive lysis buffer (Promega) and luciferase activity was measured with the Dual-Luciferase Reporter Assay System (Promega) in a Synergy 2 plate reader (BioTek Instruments, Gen5 software version 1.11) in technical triplicates from which the mean value was calculated. These mean luciferase values were determined for three biological replicates from which the mean value was reported and standard deviations, as well as statistical significance was determined. Data were processed in Microsoft Excel and plotted using GraphPad Prism.

In case of codepletion of two proteins, the first siRNA transfection contained 40 nM total siRNA (20 nM of each siRNA) and the second transfection 60 nM siRNA (40 nM INTS10, INTS13, INTS14, and 20 nM co-transfected ctrl or INTS10 siRNAs).

For rescue assays, cells were seeded and transfected as above. Roughly 24 h later, cells were reseeded at 4.7 × 10^5^ cells per well and the next day transfected again with 20 nM siRNAs. The following day, transfection mixtures contained 20 nM siRNAs, 0.4 µg U7-RLuc reporter plasmid, 0.2 µg pEGFP-N3-FLuc, and either 0.013 µg pCIneo-λN-HA-MBP plus 0.387 µg empty pcDNA3.1 vector (Invitrogen) or 0.4 µg pCIneo-λN-HA-INTS13 (wt or mutants). These mixtures were transfected using Lipofectamine 2000 according to manufacturer’s instructions. Luciferase assays were carried out as above. Expression levels of depleted and transfected proteins were checked by Western blotting.

To test the influence of HIV1-Tat on the HIV1-TAR reporter, cells were seeded as above and 24 h later transfected with 0.4 µg HIV1-TAR reporter plasmid, 0.2 µg pEGFP-N3-FLuc, and 0.2 µg pCIneo-λN-HA-MBP or pCIneo-λN-HA-HIV1-Tat using Lipofectamine 2000. Medium was changed the next day and cells harvested for luciferase assays 48 h after transfection.

### Immunofluorescence analysis

To check cellular localization of endogenous and transiently transfected INTS13 (wt and mutants) HeLa cells were seeded on circular cover slips in six-well plates and transfected as for the rescue assay, however omitting the luciferase plasmids in the second transfection. Cells were washed 5 min in 1x phosphate buffered saline (PBS) and fixed with ice cold methanol for 10 min at −20 °C. After a brief wash in 1xPBS containing 0.01% Triton-X 100 and blocking for 1 h with 10% goat serum in 2% BSA/PBS, primary antibodies diluted in blocking solution were added to the cells and incubated for 2 h. Cells were washed three times with 2% BSA/PBS before 30 min incubation with secondary antibodies diluted in blocking solution. Then cells were again washed three times with 2% BSA/PBS, once with 1xPBS, 0.1% Triton X-100, 0.02% SDS, and fixed using 4% PFA. After another wash with 1xPBS, cells were stained with Hoechst (0.5 mg/L in PBS), washed with 1xPBS and mounted in VectaShield (Vector Laboratories) onto microscope slides. Confocal images were recorded with a Zeiss 780 upright laser scanning confocal microscope and a ×63 oil objective (ScopeM, ETH Zürich).

### Statistics and reproducibility

Apart from the structural and XL–MS studies, all cell-based experiments were carried out at least three times in biological replicates, and all binding assays using purified components were performed in three independent experiments. For all quantitative measurements statistical significance of replicates was analyzed with GraphPad Prism using an unpaired, two-tailed *t*-test (Holm–Sidak method) with *p*-values below 0.05 considered significant.

### Reporting summary

Further information on research design is available in the Nature Research Reporting Summary linked to this article.

## Supplementary information

Supplementary Information

Peer Review File

Reporting Summary

Description of Additional Supplementary Files

Supplementary Data 1

Supplementary Data 2

## Data Availability

Coordinates of the crystal structure have been deposited at the Protein Data Bank under accession number 6SN1. Crosslinking mass spectrometry data is available from the proteomics data repository PRIDE^72^ with accession numbers PXD015682 (INTS13–INTS14 complex) and PXD017996 (INTS4–INTS9–INTS11–INTS13 CMBM complex). Uncropped scans of all gels and Western blots are shown in Supplementary Fig. 9, raw and processed data of all plots are included in a Source Data file. Source data are provided with this paper.

## Source data

Source Data
