# Supplementary material for: INTS10–INTS13–INTS14 form a functional module of Integrator that binds nucleic acids and the cleavage module
Source: Nat Commun. 2020 Jul 9;11:3422. doi: 10.1038/s41467-020-17232-2 (PMC7347597; doi:10.1038/s41467-020-17232-2)
Supplement: Supplementary file 1 — Supplementary Information [file 41467_2020_17232_MOESM1_ESM.pdf]

Supplementary Information for

INTS10-INTS13-INTS14 form a functional module of Integrator that binds nucleic acids and the cleavage module

Kevin Sabath[1], Melanie L. Stäubli[1], Sabrina Marti[1], Alexander Leitner[2], Murielle Moes[1], Stefanie Jonas[1]*

1 Institute of Molecular Biology and Biophysics, ETH Zurich, Switzerland
2 Institute of Molecular Systems Biology, ETH Zurich, Switzerland

*Corresponding author: stefanie.jonas@mol.biol.ethz.ch

Supplementary Table 1

Data collection and refinement statistics

DATA SET	INTS13-INTS14 (native)	INTS13-INTS14 (S-SAD)	INTS13-INTS14 (Ta)	INTS13-INTS14 (Ta)	INTS13-INTS14 (Au)	INTS13-INTS14 (Au)	INTS13-INTS14 (Au)
			**hr**	**pk**	**hr**	**pk**	**infl**
Space group	$P2_12_12$	$P2_12_12$	$P2_12_12$	$P2_12_12$	$P2_12_12$	$P2_12_12$	$P2_12_12$
Unit cell							
dimensions (a, b, c) (Å)	190.99, 115.35, 147.82	190.13, 155.31, 148.30	191.46, 115.30, 148.07	190.77, 115.43, 148.10	190.99, 115.35, 147.82	190.99, 115.35, 147.82	190.99, 115.35, 147.82
angles (α, β, γ) (°)	90, 90, 90	90, 90, 90	90, 90, 90	90, 90, 90	90, 90, 90	90, 90, 90	90, 90, 90
**DATA COLLECTION**[a]							
Wavelength	1.0000	2.0664	1.2423	1.2548	1.0282	1.0368	1.0400
Resolution range, (Å)	45.47-2.54 (2.63-2.54)	49.43-3.00 (-3.00)	49.39-3.30 (3.42-3.30)	49.38-3.30 (3.42-3.30)	48.24-3.60 (3.73-3.60)	48.24-3.60 (3.73-3.60)	47.71-3.60 (3.73-3.60)
R_{meas}, %	14.9 (380.6)		28.1 (266)	38.2 (446)	55.7 (124)	72.5 (140)	79.6 (149)
R_{pim}, %	4.0 (101.2)		4.4 (41.3)	6.0 (69.2)	8.9 (20.6)	11.6 (23.4)	21.9 (42.4)
Completeness, %	99.98 (99.82)		99.94 (99.86)	99.99 (99.96)	99.50 (98.01)	99.50 (98.19)	99.60 (97.66)
Mean $I/\sigma(I)$	16.2 (0.6)		15.6 (2.0)	11.3 (1.2)	13.0 (3.4)	11.5 (2.9)	6.9 (1.6)
Unique reflections	107565 (10595)		49973 (4900)	49887 (4885)	38334 (3689)	38334 (3696)	38369 (3676)
Multiplicity	13.7 (13.9)		39.8 (40.9)	39.8 (41.1)	39.5 (36.3)	39.5 (35.6)	13.2 (12.2)
$CC_{1/2}$	99.9 (35.4)		99.9 (78.9)	99.9 (53.6)	99.9 (92.5)	99.9 (91.7)	99.9 (78.6)
Wilson B	55.76		85.23	88.42	33.12	28.32	25.87
**REFINEMENT**							
Data range, (Å)	45.47-2.54						
R_{cryst}, %	0.2241						
R_{free}, %	0.2437						
Number of atoms per asymmetric unit							
all atoms	8015						
protein	7895						
ligand	30						
water	90						
Average B-factor, (Å^2)							
all atoms	81.83						
protein	81.61						
ligand	191.15						
water	64.81						
Ramachandran plot							
favored regions, %	96.51						
disallowed regions, %	0.20						
Rmsd from ideal geometry							
bond lengths, (Å)	0.002						
bond angles, (°)	0.481						

[a]Values in parentheses are for highest-resolution shell.

[b]R_{pim} gives the precision of averaged intensities and is a better indicator for data quality in highly redundant datasets than R_{merge}, which penalizes redundancy[4].

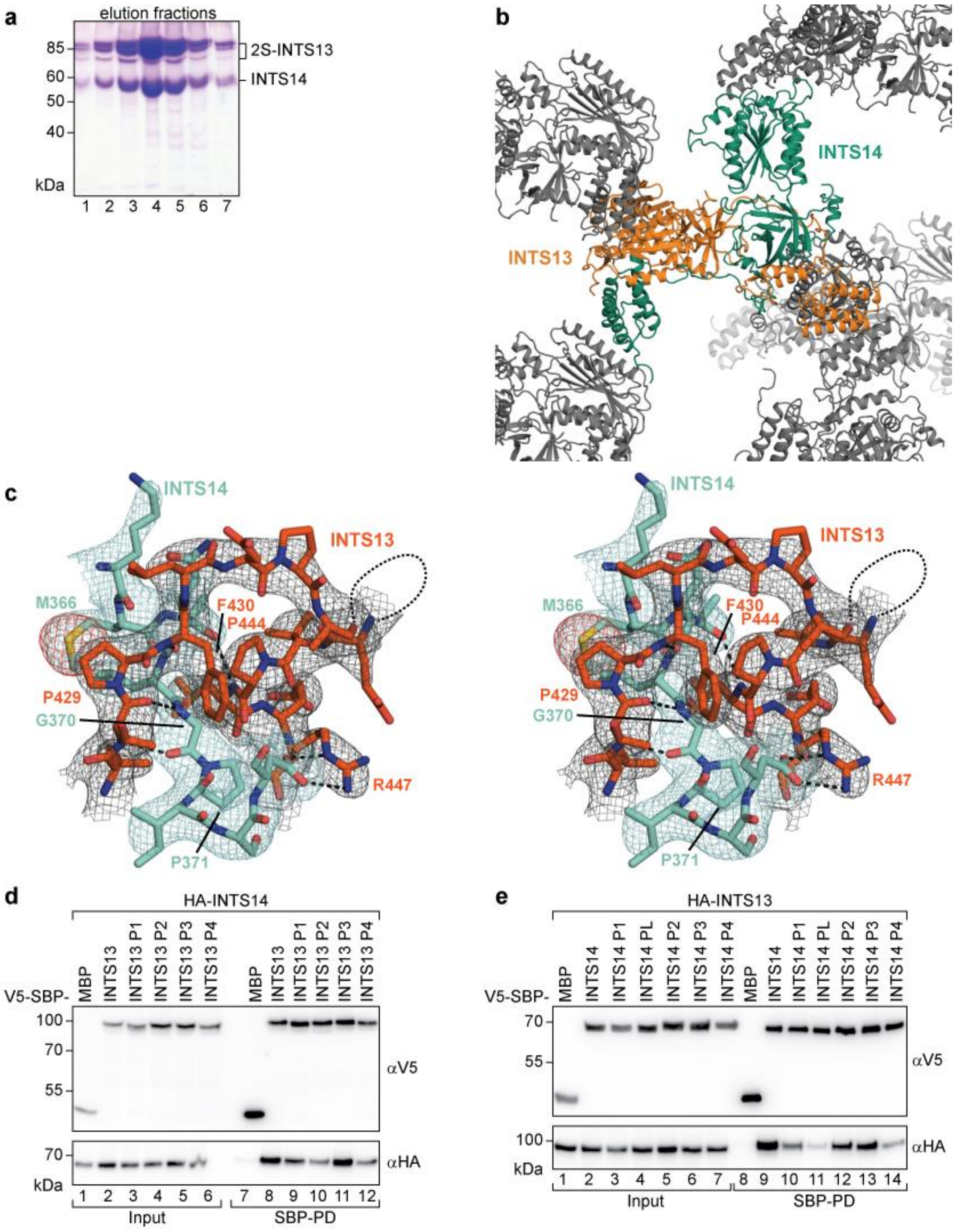

Supplementary Figure 1. INTS13-INTS14 form a well defined and stable complex.
a Copurification of 2S-INTS13 and INTS14 expressed in insect cells. The complex remains intact over three purification steps. For INTS13 the full length protein and a C-terminally truncated fragment are visible, which becomes progressively prominent over time. **b** Crystal packing of the INTS13-INTS14 complex. Symmetry mates contacting the complex are shown in grey. Contacts are solely mediated by the VWA and α-helical domains of both proteins. The Linker region of either protein is not involved in crystal packing. **c** Stereo view of the electron density $2F_o$-F_c map of the INTS13-INTS14 crystal structure contoured at 1.5σ. The density surrounding the interlink is shown as a grey mesh with the INTS13 model in orange and INTS14 in light blue. The anomalous sulfur density in the same area is contoured at 5.0σ and shown as a red mesh. **d** Coprecipitation of HA-INTS14 with single binding patch mutants of V5-SBP-INTS13 transfected into HEK293T cells. V5-SBP-MBP is the negative control. Inputs (αV5-blot 1%, αHA-blot 0.38%) and bound fractions (αV5-blot 7.5%, αHA-blot 20%) were analyzed

by Western blotting. **e** Single binding patch mutants of V5-SBP-INTS14 were tested for coprecipitation of HA-INTS13 from HEK293T cells. Inputs (αV5-blot 1%, αHA-blot 0.88%) and bound fractions (αV5-blot 4%, αHA-blot 17.5%) were analyzed by Western blotting.

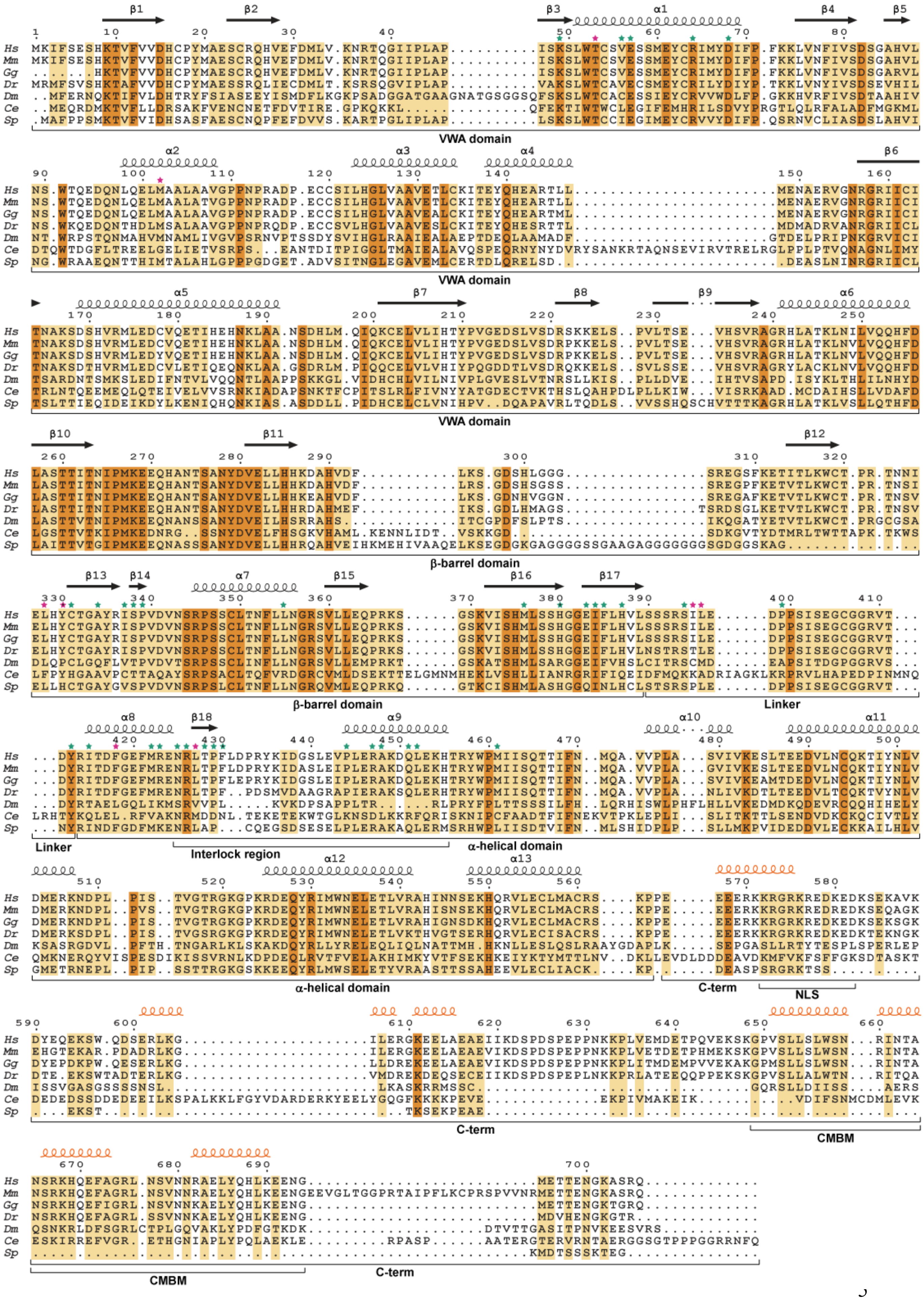

Hs
Mm
Gg
Dr
Dm
Ce
Sp
VWA domain
β-barrel domain
Linker
Interlock region
α-helical domain
C-term
NLS
CMBM

Supplementary Figure 2. INTS13 residues involved in INTS14 binding are conserved. Sequence alignment of INTS13 orthologs from *Homo sapiens* (*Hs*), *Mus musculus* (*Mm*), *Gallus gallus* (*Gg*), *Danio rerio* (*Dr*), *Drosophila melanogaster* (*Dm*), *Caenorhabditis elegans* (*Ce*), *Strongylocentrotus purpuratus* (*Sp,* Purple urchin). Residues with complete conservation and 70% similarity are highlighted in orange and pale yellow, respectively. Secondary structure elements as observed in the INTS13-INTS14 structure are denoted in black above the alignment, while elements in the C-terminus as predicted by JPred[1] are shown in orange. Protein domains and important regions or motifs are marked below the alignment. Green stars indicate amino acids that are part of the interface with INTS14, and pink stars highlight residues that were mutated in this study.

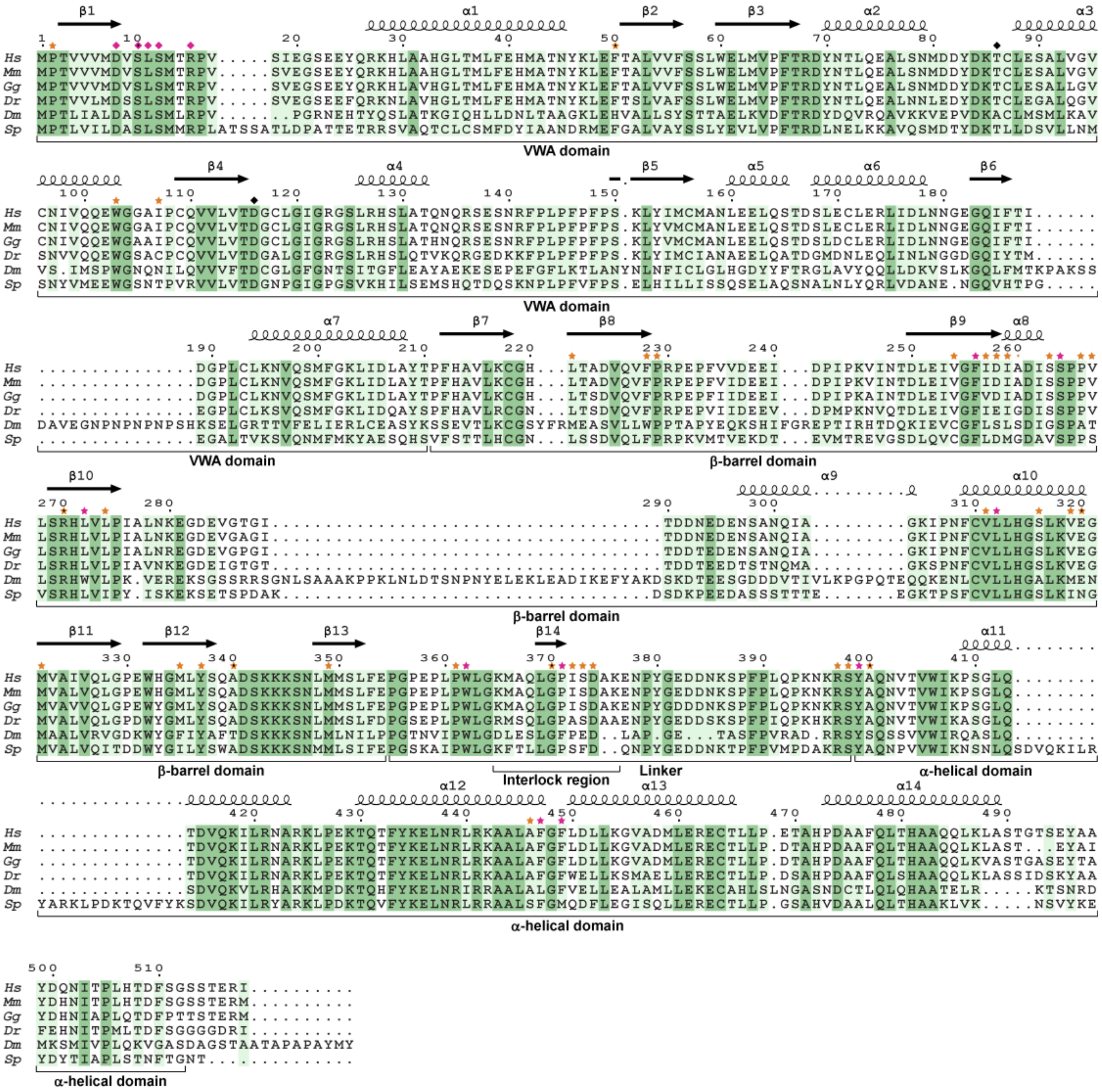

Supplementary Figure 3. INTS14 residues involved in INTS13 binding are conserved. Sequence alignment of INTS14 orthologs. Residues with complete conservation and 70% similarity are highlighted in darker and light green, respectively. Orange stars indicate amino acids that are part of the interface with INTS13, while pink stars highlight interface residues that were mutated. Diamonds (black/pink) mark residues that constitute the MIDAS pocket and pink diamonds indicate MIDAS amino acids that were mutated. Abbreviations and labelling as in Supplementary Figure 2.

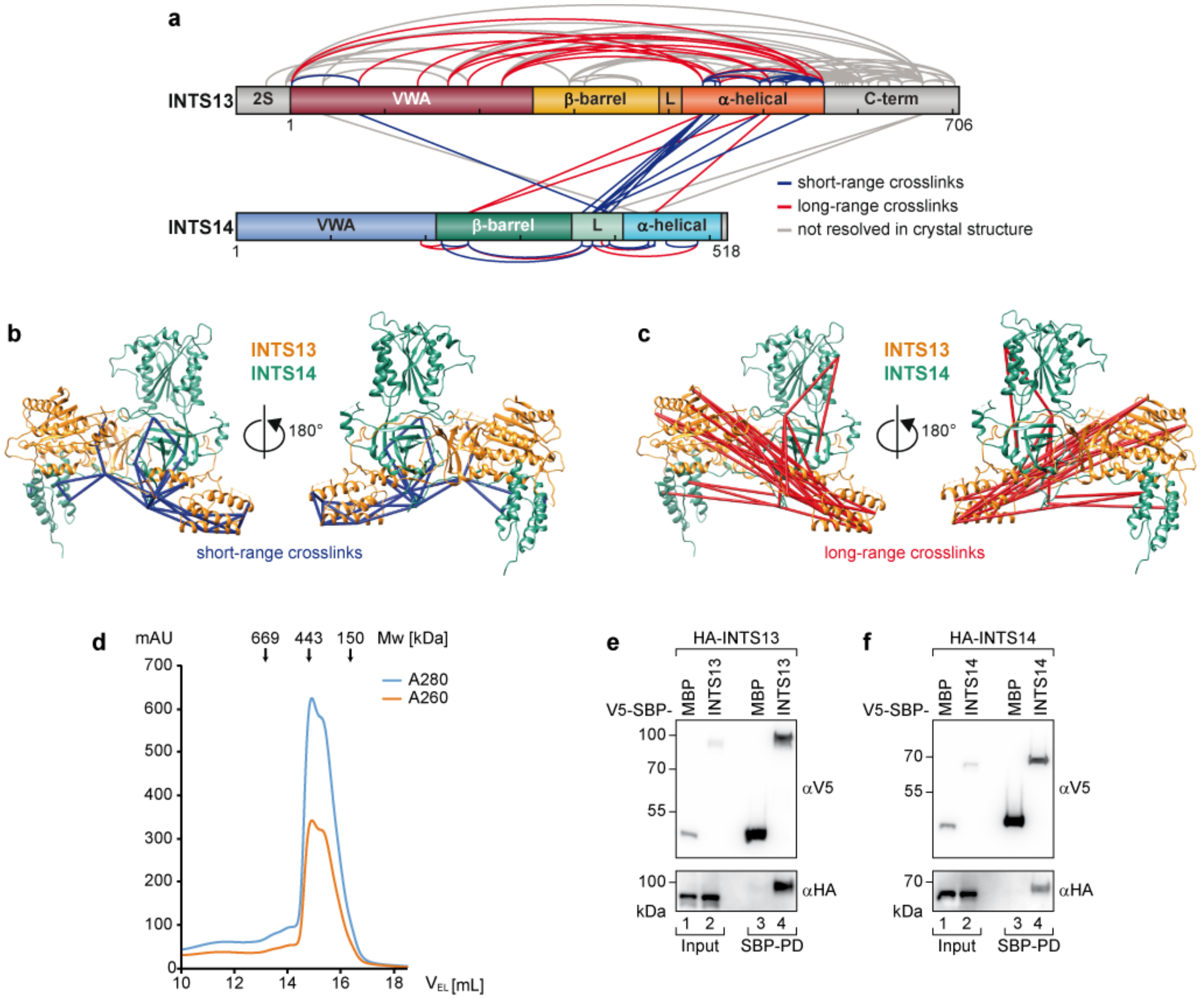

Supplementary Figure 4. The INTS13-INTS14 complex interacts with itself *in vitro* and in cells.

a DSS crosslinks between lysine sidechains in the INTS13-INTS14 complex. Colored lines show crosslinks of residues that are part of the crystal structure, while grey lines represent crosslinks between residues not visualized in the structure. Blue lines indicate crosslinks with a Cα distance below 30 Å, that are likely resulting from DSS-links within one INTS13-INTS14 complex. Red lines represent long range crosslinks, that are likely the result of complex oligomerization. **b, c** Mapping of the short (blue) and long (red) range crosslinks onto the INTS13-INTS14 structure, indicating the links as lines between Cα atoms of the respective Lys residues. **d** Size exclusion chromatography profile of INTS13-INTS14. The complex elutes as a double peak corresponding to a dimer and a trimer of the heterodimeric complex ($M_{w,monomer}$ 143 kDa, $M_{w,dimer}$ 286 kDa, $M_{w,trimer}$ 429 kDa). Source data are provided as a Source Data file. **e**, **f** Co-precipitation of HA-INTS13 with V5-SBP-INTS13 (**e**) and of HA-INTS14 with V5-SBP-INTS14 (**f**) from HEK293T cells, indicates self-interaction in cells. V5-SBP-MBP served as a control for background binding. Inputs (αV5-blot 1%, αHA-blot 0.38%) and bound fractions (αV5-blot 7.5%, αHA-blot 20%) were analyzed by Western blotting.

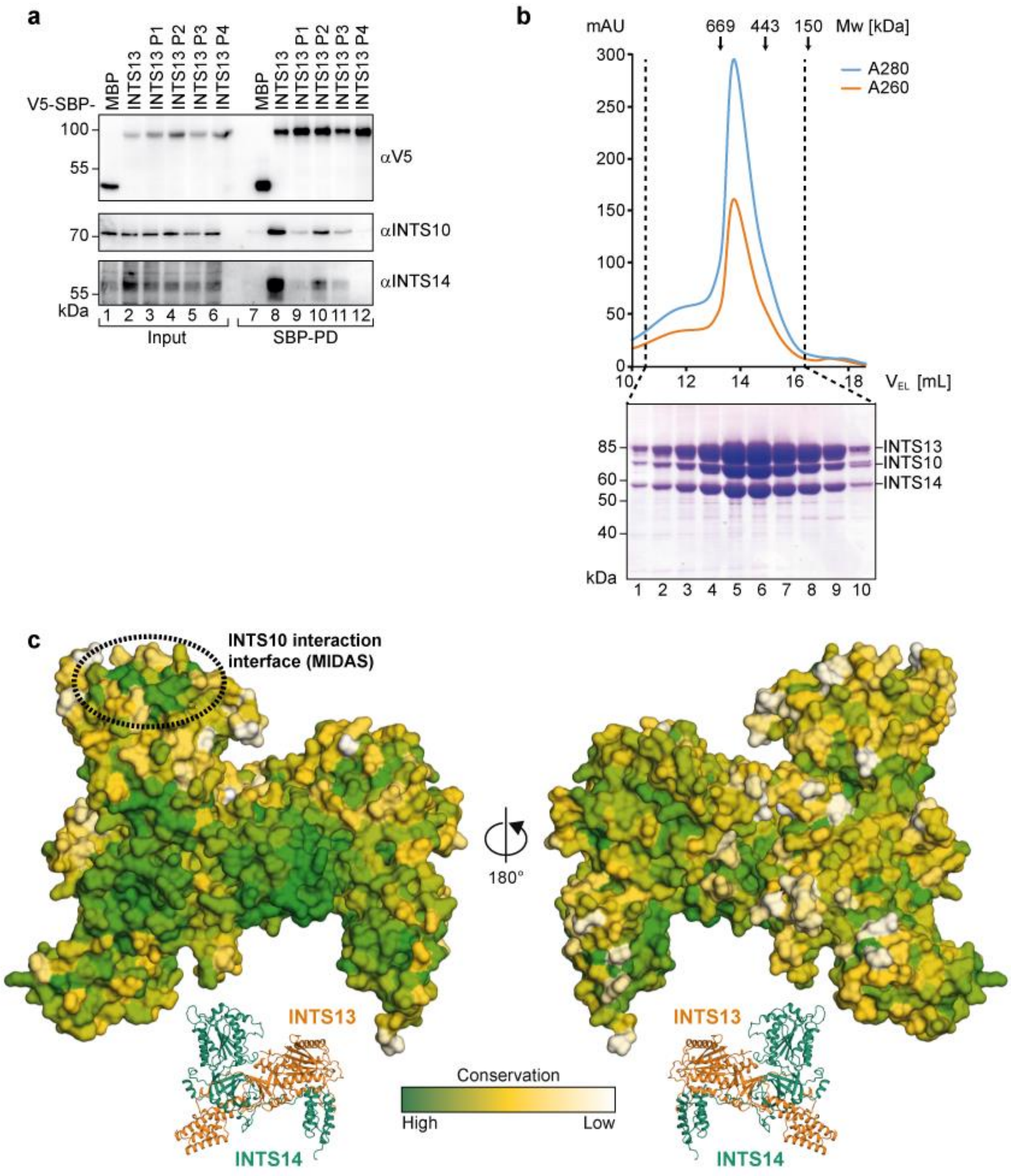

Supplementary Figure 5. INTS10 forms a stable complex with INTS13-INTS14.
a Coprecipitation of endogenous INTS14 and INTS10 with single binding patch mutants of V5-SBP-INTS13 expressed in HEK293T cells. V5-SBP-MBP served as a control for unspecific binding. Inputs (αV5-blot 1%, endog.-blots 0.38%) and bound fractions (αV5-blot 5%, endog.-blots 17.5%) were analyzed by Western blotting. **b** The INTS10-2S-INTS13-INTS14 complex copurifies over three purification steps. During size exclusion chromatography the complex elutes in fractions corresponding to a dimeric heterotrimer ($M_{w,monomer}$ 226 kDa, $M_{w,dimer}$ 452 kDa). Source data are provided as a Source Data file. **c** Surface of the INTS13-INTS14 heterodimer colored according to surface conservation in a gradient from green (high conservation) over yellow to white (low conservation) as calculated by ConSurf[2]. The small cartoon representations below show the orientation of the structures. The highly conserved surface patch on top of the INTS14 VWA domain corresponding to the MIDAS pocket is marked with a dotted line.

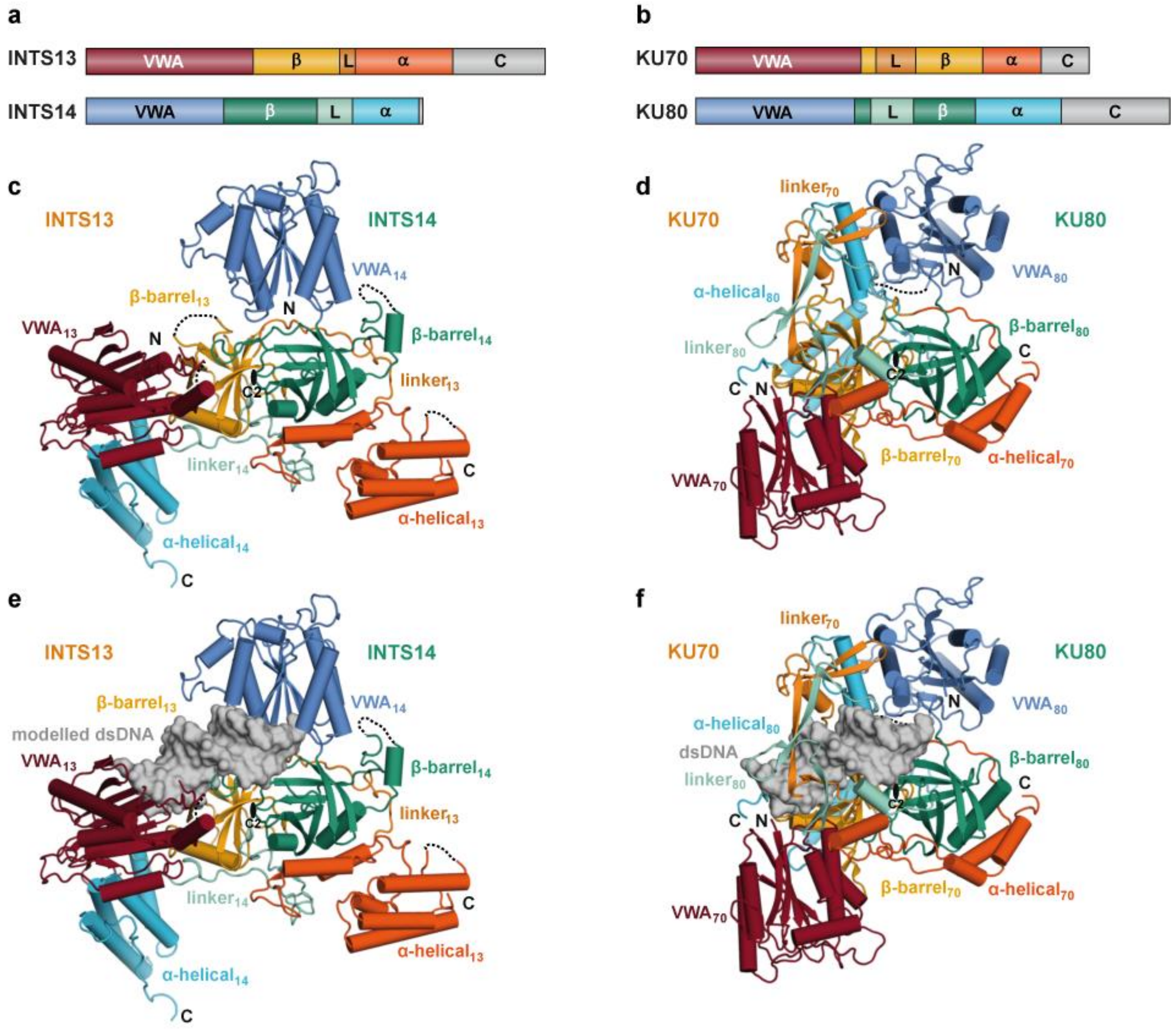

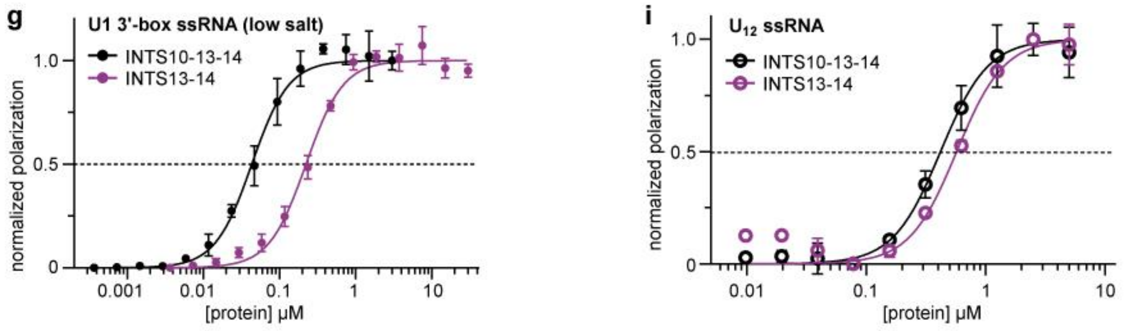

h

U1 3'-box (low salt)	K_d [μM]
INTS13-14	0.223 ± 0.014
INTS10-13-14	0.043 ± 0.002

j

U_{12} ssRNA	K_d [μM]
INTS13-14	0.57 ± 0.05
INTS10-13-14	0.42 ± 0.02

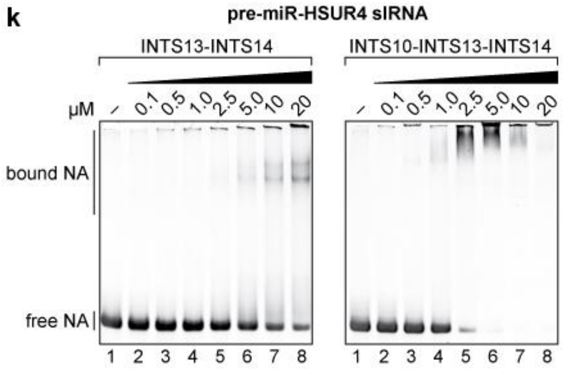

Supplementary Figure 6. The INTS13-INTS14 complex shares structural homology with the DNA repair complex Ku70-Ku80.

a, b Domain schemes of INTS13, INTS14 and Ku70, Ku80 show a similar domain arrangement for all four proteins. Corresponding domains are shown in the same color. **c**, **d** The two complexes share overall structural similarities. Coloring of the domains as in **a**, **b**. Ku70-Ku80 (PDB-ID: 1JEY [3]) is more symmetrical than INTS13-INTS14 with an almost perfect two-fold rotational symmetry. The position of the pseudosymmetry C2 axis is indicated with a black ellipse. Structures were superposed based on their β-barrel domains and shown side by side. **e, f** dsDNA (grey) co-crystallized with the Ku70-Ku80 heterodimer is modelled onto the INTS13-INTS14 structure, where it shows strong clashes with the INTS13 VWA domain, indicating that analogous dsDNA binding is unlikely. **g-j** Fluorescence polarization assays of 5'-FAM-U1 3'-box ssRNA under low salt conditions (**g**, **h**) or 5'-FAM-U_{12} ssRNA (**i**, **j**) with increasing concentrations of 2S-INTS13-INTS14 (purple) or INTS10-2S-INTS13-INTS14 (black), and the extracted dissociation constants. In both graphs mean values of experimental triplicates are plotted and error bars represent standard deviations. Dissociation constants were obtained by fitting the data by linear regression, and are listed with their standard error of means. Source data are provided as a Source Data file. **k** EMSAs of INTS13-INTS14 (left panel) and INTS10-INTS13-INTS14 (right panel) with pre-miR-HSUR4 stem loop RNA. Increasing protein concentrations were incubated with the respective nucleic acid, resolved on a native acrylamide gel and stained with a fluorophore.

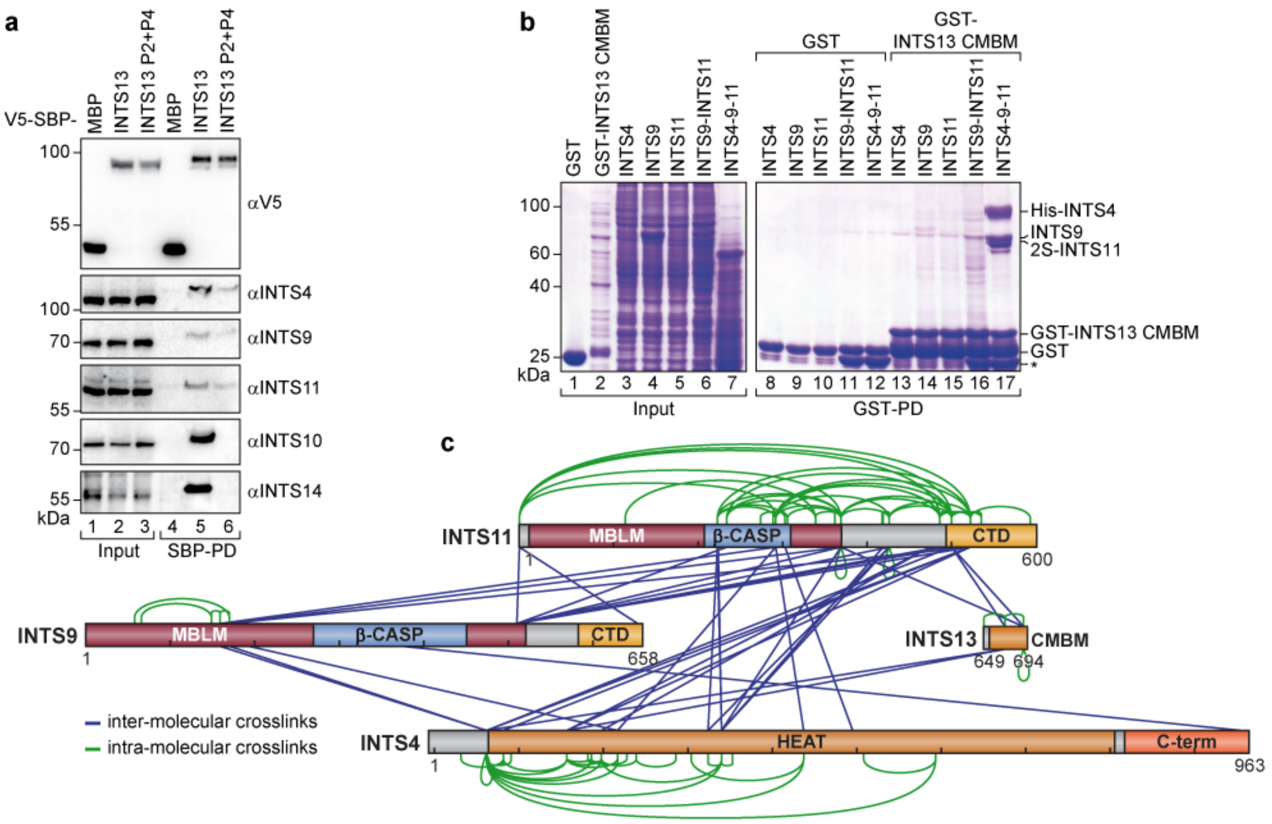

Supplementary Figure 7. All subunits of the cleavage module are required for INTS13 binding.

a Co-precipitation of endogenous INT subunits with V5-SBP-INTS13 wild type and mutant. V5-SBP-MBP is included as a control. Inputs (αV5-blot 1%, endog.-blots 0.38%) and bound fractions (αV5-blot 4%, endog.-blots 20%) were analyzed by Western blotting. **b** Co-purification of INT cleavage module components from insect cell extracts with GST-INTS13 CMBM from *E.coli* lysates. A Coomassie stained gel is shown. GST was used as a negative control. The asterisk marks the putative GST from insect cells that also binds to GSH-beads. **c** DSS crosslinks between Lys residues of the cleavage module (INTS4-INTS9-INTS11) and the CMBM of INTS13 as determined by XL-MS. Crosslinks within one protein chain are shown in green, while crosslinks between different proteins are shown in blue. Every 100th residue is marked with a tick. Abbreviations: MBLM – metallo β-lactamase, β-CASP - CPSF/Artemis/Snm1/Pso2 like domain, CTD – C-terminal domain, HEAT – Huntingtin/elongation factor 3/protein phosphatase 2A/TOR1 repeats.

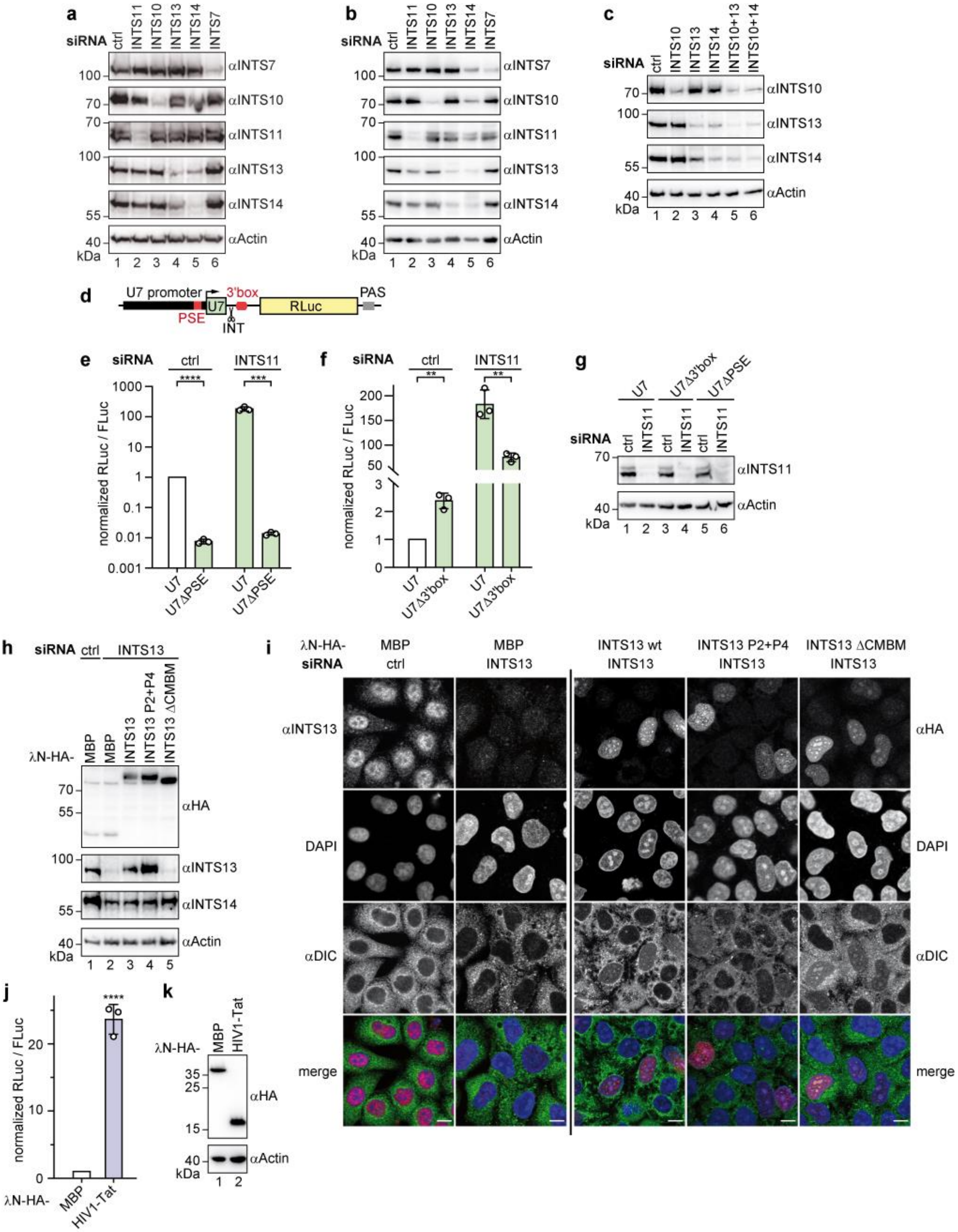

Supplementary Figure 8. Validation of reporter constructs and correct localization of INTS13 mutants.

a-c Western blots of depletions corresponding to Fig. 6c (**a**), 6e (**b**), and 6f (**c**). β-actin served as loading control. **d** Schematic of U7 reporter constructs used in **e** and **f** with proximal sequence element (PSE) of the promoter and processing signal (3'-box) highlighted in red. **e** Luciferase

assay with full U7 reporter or one without PSE in control cells or cells depleted of INTS11. A CMV-promoter driven FLuc plasmid was cotransfected for normalization of transfection efficiency. Luciferase levels relative to the unmodified U7 reporter in control cells are shown on a logarithmic scale. All bar graphs depict mean values from biological triplicates shown together with individual values as dots, and error bars representing standard deviations. Statistical significance was determined by unpaired, two-tailed t-test: not significant (ns) $p \geq 0.05$; significant * $p \leq 0.05$, ** $p \leq 0.01$, *** $p \leq 0.001$, **** $p \leq 0.0001$. **f** Luciferase assays comparing the full U7 reporter to one without the 3'-box in control cells or cells depleted of INTS11. Normalization, plotting and statistics as in **e**. **g** Western blots of depletions corresponding to **e** and **f**. β-actin served as loading control. **h** Western blots corresponding to Fig. 6g. β-actin served as loading control. Of note, because the monoclonal anti-INTS13 antibody was raised against the INTS13 C-terminus, it does not recognize the INTS13 ΔCMBM construct (lane 5). **i** Immunofluorescence staining of endogenous INTS13 or transfected constructs (red) from the rescue assay in Fig. 6g. Endogenous INTS13 localizes in the nucleus, since only the nuclear signal decreases upon siRNA treatment. All transfected HA-tagged INTS13 constructs (wt or mutants) localize to nuclei. DAPI (blue) visualizes the nucleus, and anti-dynein intermediate chain (DIC, green) the cytoplasm. Scale bar 10 µm. **j** Verification of the HIV-1 TAR reporter by cotransfection of HIV-1 Tat, which leads to pause release of RNAPII from the TAR element and transcription elongation. MBP served as control, and a CMV-driven FLuc plasmid as control to normalize for transfection efficiencies. Plotting and statistics as in **e**. **k** Western blot of transfected proteins from **j**. β-Actin served as loading control. Source data are provided as a Source Data file.

Supplementary Figure 9. Uncropped images of all gels and Western blots.

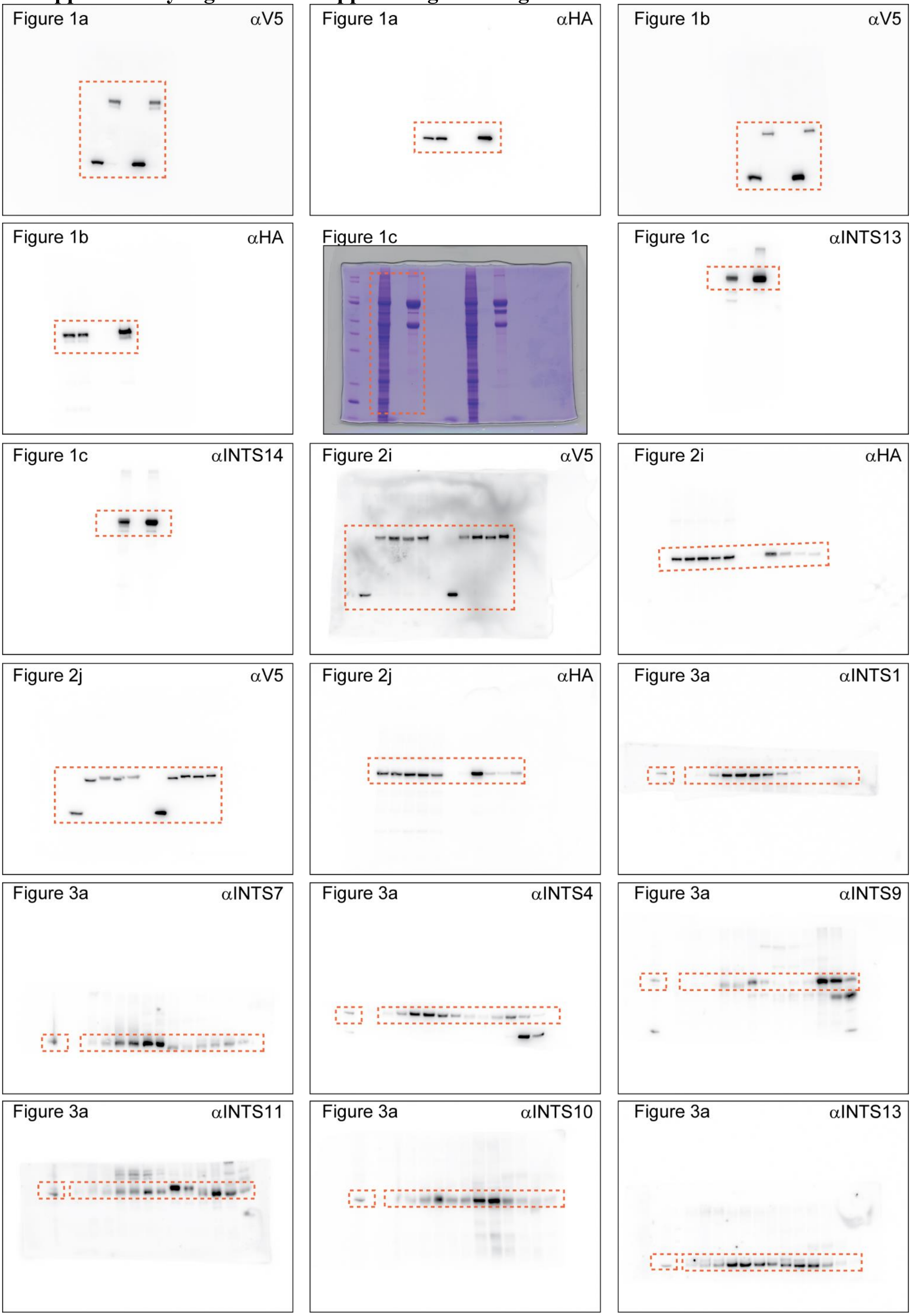

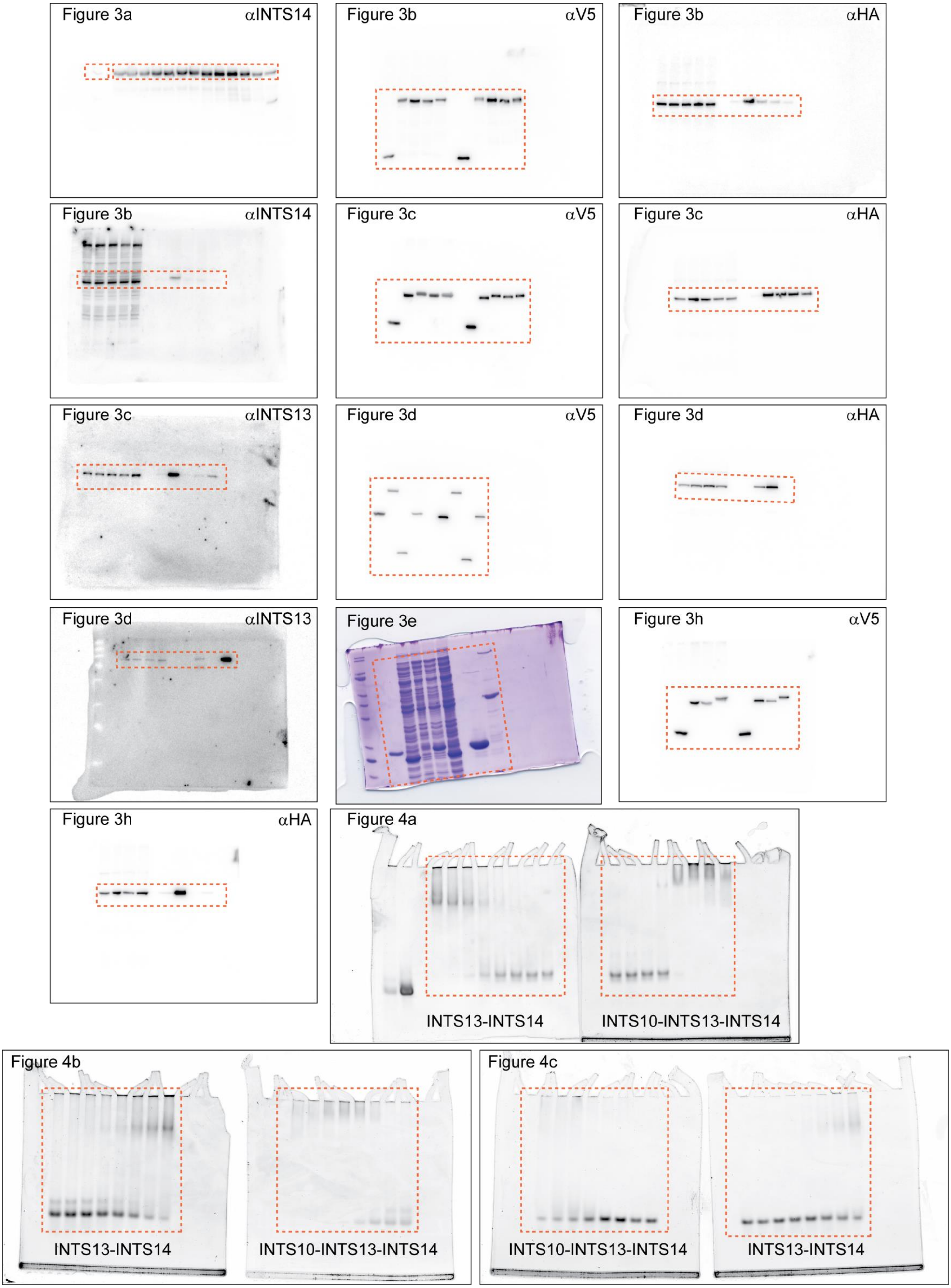
Figure 3a
αINTS14
Figure 3b
αV5
Figure 3b
αHA
Figure 3b
αINTS14
Figure 3c
αV5
Figure 3c
αHA
Figure 3c
αINTS13
Figure 3d
αV5
Figure 3d
αHA
Figure 3d
αINTS13
Figure 3e
Figure 3h
αV5
Figure 3h
αHA
Figure 4a
INTS13-INTS14
INTS10-INTS13-INTS14
Figure 4b
INTS13-INTS14
INTS10-INTS13-INTS14
Figure 4c
INTS10-INTS13-INTS14
INTS13-INTS14

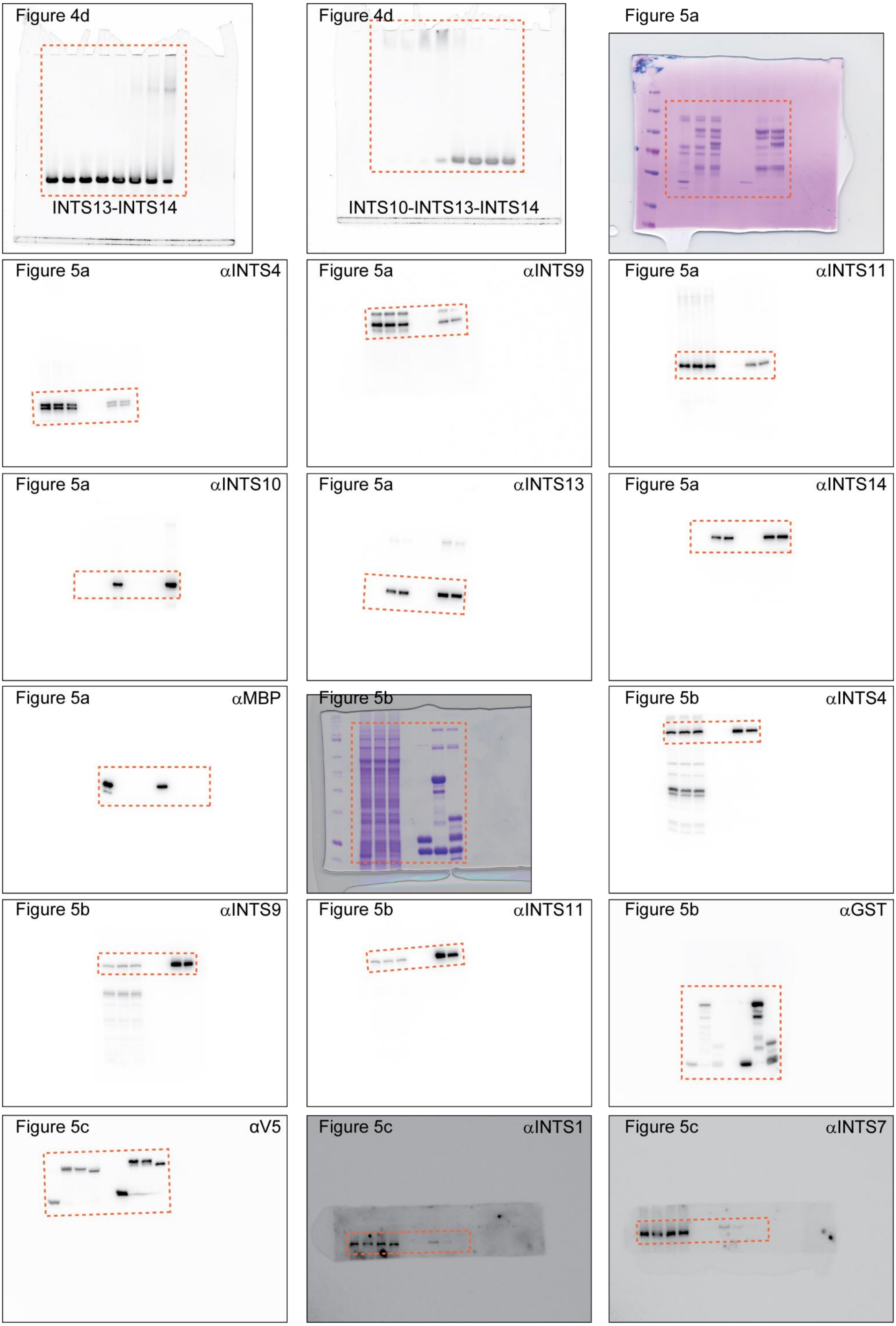
Figure 4d
INTS13-INTS14
Figure 4d
INTS10-INTS13-INTS14
Figure 5a
Figure 5a αINTS4
Figure 5a αINTS9
Figure 5a αINTS11
Figure 5a αINTS10
Figure 5a αINTS13
Figure 5a αINTS14
Figure 5a αMBP
Figure 5b
Figure 5b αINTS4
Figure 5b αINTS9
Figure 5b αINTS11
Figure 5b αGST
Figure 5c αV5
Figure 5c αINTS1
Figure 5c αINTS7

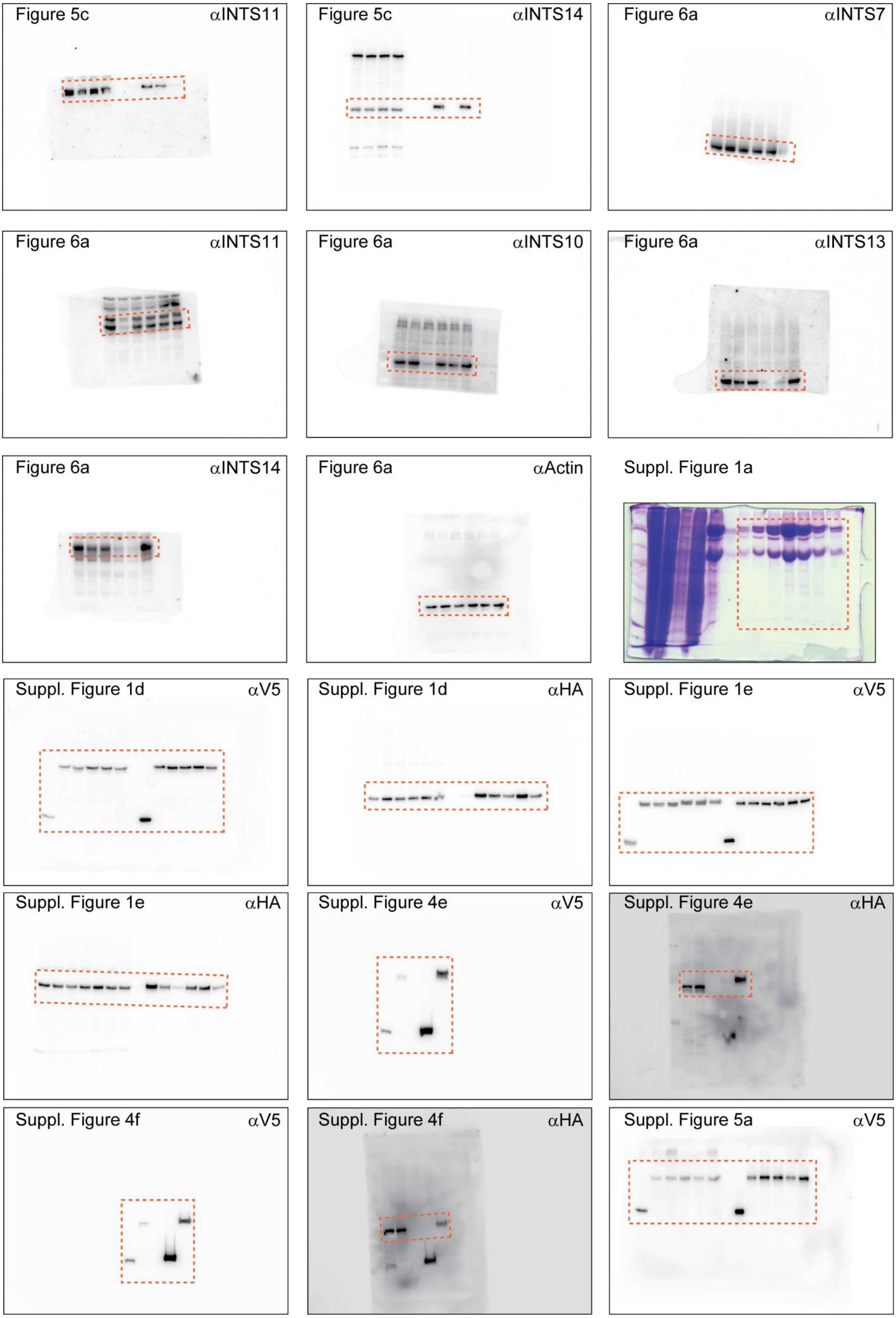
Figure 5c αINTS11
Figure 5c αINTS14
Figure 6a αINTS7
Figure 6a αINTS11
Figure 6a αINTS10
Figure 6a αINTS13
Figure 6a αINTS14
Figure 6a αActin
Suppl. Figure 1a
Suppl. Figure 1d αV5
Suppl. Figure 1d αHA
Suppl. Figure 1e αV5
Suppl. Figure 1e αHA
Suppl. Figure 4e αV5
Suppl. Figure 4e αHA
Suppl. Figure 4f αV5
Suppl. Figure 4f αHA
Suppl. Figure 5a αV5

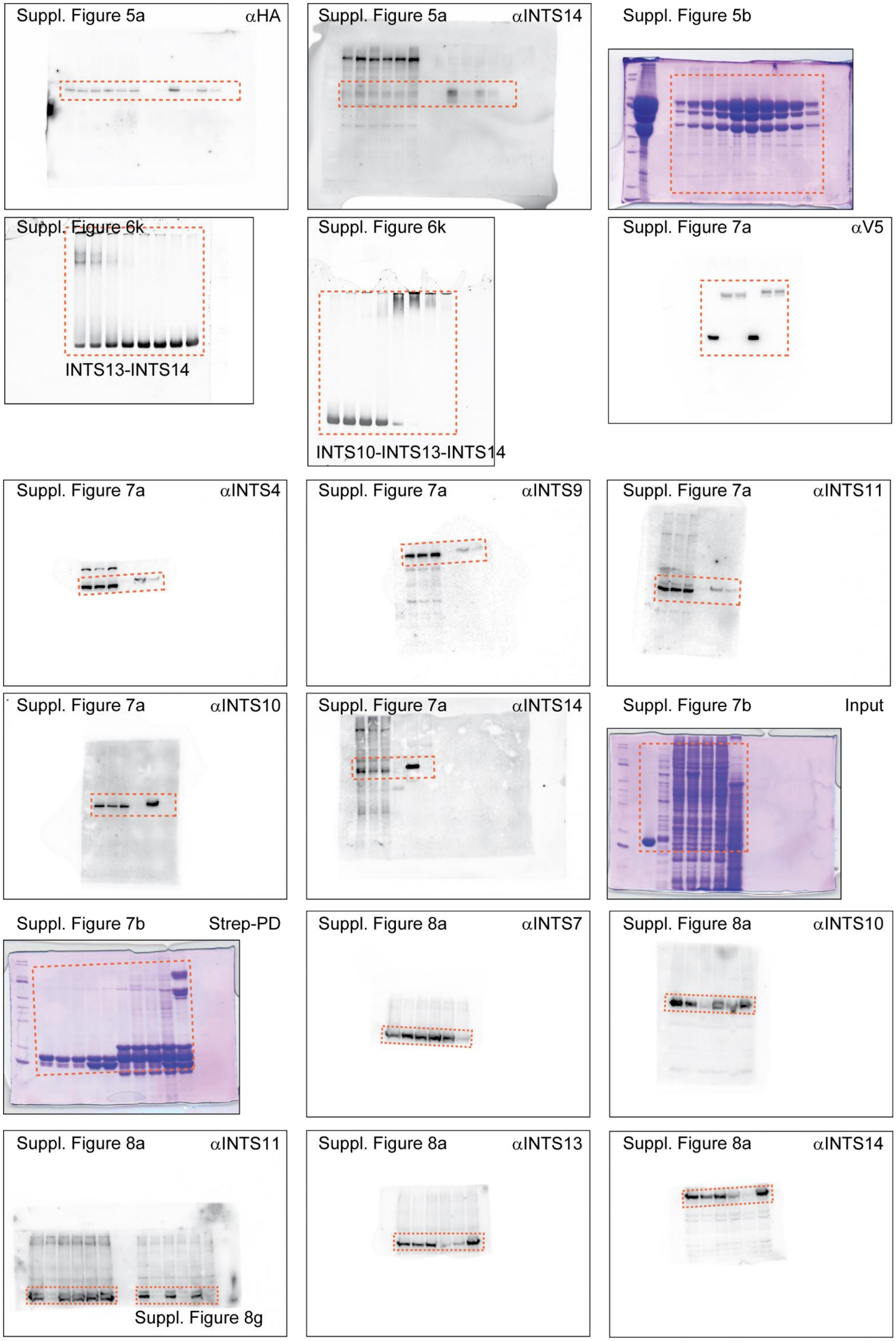

Suppl. Figure 5a
αHA
Suppl. Figure 5a
αINTS14
Suppl. Figure 5b
Suppl. Figure 6k
INTS13-INTS14
Suppl. Figure 6k
INTS10-INTS13-INTS14
Suppl. Figure 7a
αV5
Suppl. Figure 7a
αINTS4
Suppl. Figure 7a
αINTS9
Suppl. Figure 7a
αINTS11
Suppl. Figure 7a
αINTS10
Suppl. Figure 7a
αINTS14
Suppl. Figure 7b
Input
Suppl. Figure 7b
Strep-PD
Suppl. Figure 8a
αINTS7
Suppl. Figure 8a
αINTS10
Suppl. Figure 8a
αINTS11
Suppl. Figure 8g
Suppl. Figure 8a
αINTS13
Suppl. Figure 8a
αINTS14

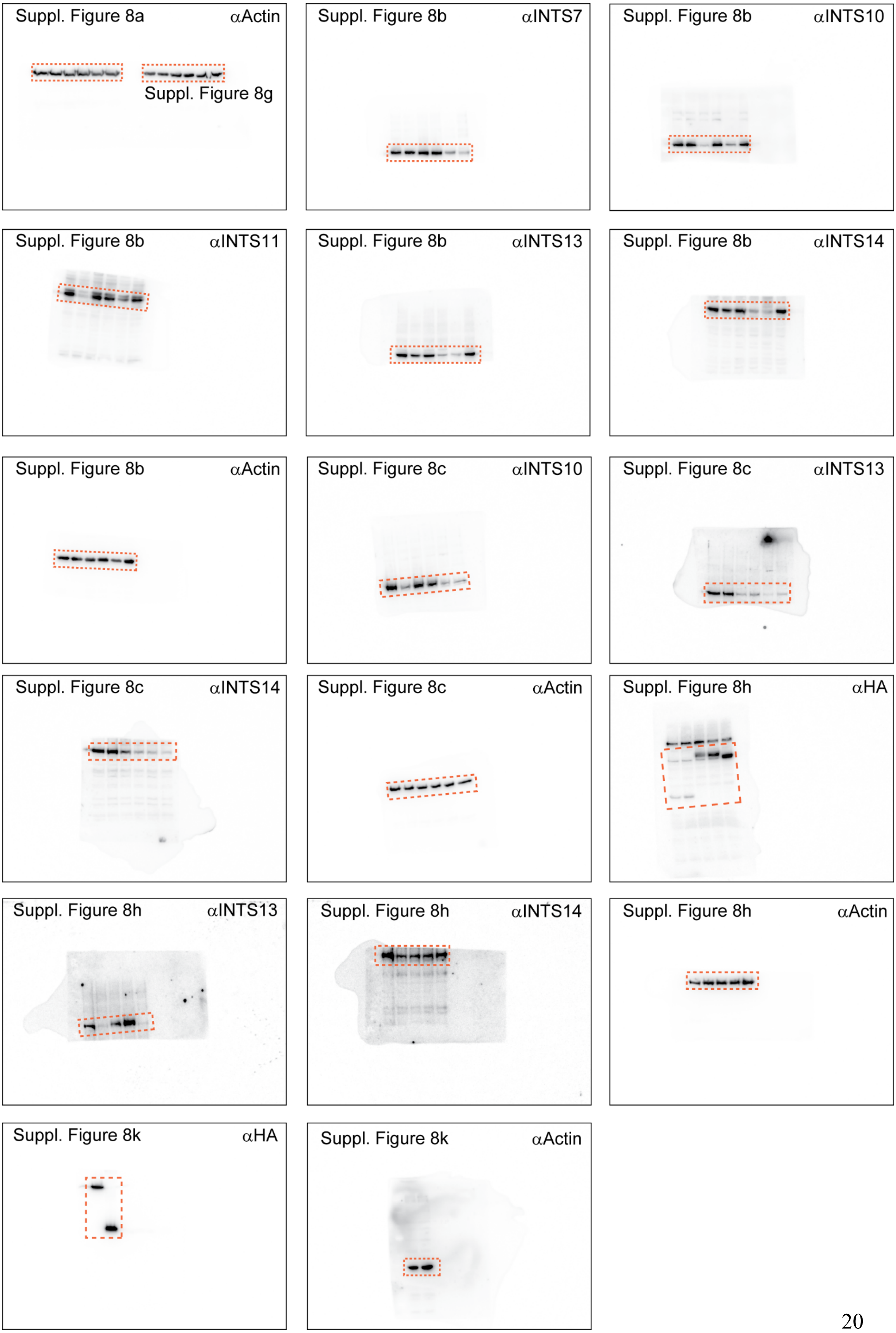
Suppl. Figure 8a
αActin
Suppl. Figure 8g
Suppl. Figure 8b
αINTS7
Suppl. Figure 8b
αINTS10
Suppl. Figure 8b
αINTS11
Suppl. Figure 8b
αINTS13
Suppl. Figure 8b
αINTS14
Suppl. Figure 8b
αActin
Suppl. Figure 8c
αINTS10
Suppl. Figure 8c
αINTS13
Suppl. Figure 8c
αINTS14
Suppl. Figure 8c
αActin
Suppl. Figure 8h
αHA
Suppl. Figure 8h
αINTS13
Suppl. Figure 8h
αINTS14
Suppl. Figure 8h
αActin
Suppl. Figure 8k
αHA
Suppl. Figure 8k
αActin

SUPPLEMENTARY REFERENCES

1. Cole, C., Barber, J. D. & Barton, G. J. The Jpred 3 secondary structure prediction server. *Nucleic Acids Res.* **36**, W197-201 (2008).
2. Ashkenazy, H. *et al.* ConSurf 2016: an improved methodology to estimate and visualize evolutionary conservation in macromolecules. *Nucleic Acids Res.* **44**, W344-350 (2016).
3. Walker, J. R., Corpina, R. A. & Goldberg, J. Structure of the Ku heterodimer bound to DNA and its implications for double-strand break repair. *Nature* **412**, 607–614 (2001).
4. Weiss, M. S. Global indicators of X-ray data quality. *J Appl Cryst* **34**, 130–135 (2001).
