# Supplementary material for: INTS10–INTS13–INTS14 form a functional module of Integrator that binds nucleic acids and the cleavage module
Source: Nat Commun. 2020 Jul 9;11:3422. doi: 10.1038/s41467-020-17232-2 (PMC7347597; doi:10.1038/s41467-020-17232-2)
Supplement: Supplementary file 3 — Reporting Summary [file 41467_2020_17232_MOESM3_ESM.pdf]

nature research

Corresponding author(s): Stefanie Jonas

Last updated by author(s): Jun 15, 2020

Reporting Summary

Nature Research wishes to improve the reproducibility of the work that we publish. This form provides structure for consistency and transparency in reporting. For further information on Nature Research policies, see Authors & Referees and the Editorial Policy Checklist .

Statistics

For all statistical analyses, confirm that the following items are present in the figure legend, table legend, main text, or Methods section.

n/a	Confirmed	
☐	☒	The exact sample size (*n*) for each experimental group/condition, given as a discrete number and unit of measurement
☐	☒	A statement on whether measurements were taken from distinct samples or whether the same sample was measured repeatedly
☐	☒	The statistical test(s) used AND whether they are one- or two-sided<br>*Only common tests should be described solely by name; describe more complex techniques in the Methods section.*
☒	☐	A description of all covariates tested
☒	☐	A description of any assumptions or corrections, such as tests of normality and adjustment for multiple comparisons
☐	☒	A full description of the statistical parameters including central tendency (e.g. means) or other basic estimates (e.g. regression coefficient) AND variation (e.g. standard deviation) or associated estimates of uncertainty (e.g. confidence intervals)
☐	☒	For null hypothesis testing, the test statistic (e.g. *F*, *t*, *r*) with confidence intervals, effect sizes, degrees of freedom and *P* value noted<br>*Give P values as exact values whenever suitable.*
☒	☐	For Bayesian analysis, information on the choice of priors and Markov chain Monte Carlo settings
☒	☐	For hierarchical and complex designs, identification of the appropriate level for tests and full reporting of outcomes
☒	☐	Estimates of effect sizes (e.g. Cohen's *d*, Pearson's *r*), indicating how they were calculated

Our web collection on statistics for biologists contains articles on many of the points above.

Software and code

Policy information about availability of computer code

Data collection	Light intensities in luciferase assays were measured in a Synergy 2 plate reader (BioTek Instruments) and Gen5 software (Version 1.11); Fluorescence polarization was determined with a CLARIOstar microplate reader (BMG Labtech) and CLARIOstar software (Version 5.40-1); Diffraction data were recorded on a PILATUS 6M detector at the PXIII beamline of the Swiss Light Source (SLS) with DA+ software (no version available); Raw mass spectrometry data were acquired using Thermo Xcalibur 4.2.28.14.
Data analysis	XDS and XSCALE (both Version Jan 26, 2018) were used to process diffraction data; SHELXD (Version 2013/2), MLPHARE in CCP4 (Version 7.0), and PHASER EP in Phenix (Version 2.8.2) were used for phasing; COOT (Version 0.8.9.2) and Phenix Refine (1.15.2-3472) were used for model building and refinement; Microsoft Excel (2016) and GraphPad Prism (Version 8.4.2) were used to normalize and fit fluorescence polarization values; MS/MS spectra were analyzed using xQuest 2.1.4. and Mascot (Version 2.5.1, MatrixScience)

For manuscripts utilizing custom algorithms or software that are central to the research but not yet described in published literature, software must be made available to editors/reviewers. We strongly encourage code deposition in a community repository (e.g. GitHub). See the Nature Research guidelines for submitting code & software for further information.

Data

Policy information about availability of data

All manuscripts must include a data availability statement. This statement should provide the following information, where applicable:

- Accession codes, unique identifiers, or web links for publicly available datasets
- A list of figures that have associated raw data
- A description of any restrictions on data availability

Coordinates of the crystal structure have been deposited at the Protein Data Bank under accession number 6SN1. Crosslinking mass spectrometry data is available from the proteomics data repository PRIDE under dataset identifiers PXD015682 and PXD017996. All uncropped gels and Western blots are included in Supplementary Fig. 9, and the raw data for all plots is included in a Source Data file.

Field-specific reporting

Please select the one below that is the best fit for your research. If you are not sure, read the appropriate sections before making your selection.

☒ Life sciences ☐ Behavioural & social sciences ☐ Ecological, evolutionary & environmental sciences

For a reference copy of the document with all sections, see nature.com/documents/nr-reporting-summary-flat.pdf

Life sciences study design

All studies must disclose on these points even when the disclosure is negative.

Sample size	n/a
Data exclusions	No data were excluded from analysis.
Replication	Reproducibility was ensured by repeating each experiment three times. All attempts at replication were successful.
Randomization	n/a
Blinding	n/a

Reporting for specific materials, systems and methods

We require information from authors about some types of materials, experimental systems and methods used in many studies. Here, indicate whether each material, system or method listed is relevant to your study. If you are not sure if a list item applies to your research, read the appropriate section before selecting a response.

Materials & experimental systems

n/a	Involved in the study	
☐	☒	Antibodies
☐	☒	Eukaryotic cell lines
☒	☐	Palaeontology
☒	☐	Animals and other organisms
☒	☐	Human research participants
☒	☐	Clinical data

Methods

n/a	Involved in the study	
☒	☐	ChIP-seq
☒	☐	Flow cytometry
☒	☐	MRI-based neuroimaging

Antibodies

Antibodies used

Primary antibodies: horseradish peroxidase linked anti-HA antibody (Roche, 12013819001), rabbit anti-INTS1 (Bethyl, A300-361A), rabbit anti-INTS4 (Bethyl Laboratories, A301-269A), rabbit anti-INTS7 (Bethyl, A300-271A), rabbit anti-INTS9 (Proteintech, 11657-1-AP), rabbit anti-INTS10 (Proteintech, 15271-1-AP), rabbit anti-INTS11 (Bethyl, A301-274A), rabbit anti-INTS13 (Bethyl, A303-575A), rabbit anti-INTS14 (Bethyl, A303-576A), goat anti-GST (GE Healthcare, 27-4577-01), mouse anti-V5 (AbD Serotec, MCA 1360), mouse anti-β-actin (Proteintech, 60008-1-Ig), mouse anti-Dynein IC (Millipore, MAB1618).

Secondary antibodies: horseradish peroxidase-linked anti-mouse IgG (Sigma, A9044), anti-rabbit IgG (Sigma, A9169-2ML), anti-goat IgG (Sigma, A8919), rat anti-HA (Roche, 11867423001), Alexa Fluor 488-labeled goat anti-mouse (Invitrogen, A1100), Alexa Fluor 568-labeled goat anti-rabbit (Invitrogen, A11011), Alexa Fluor 633-labeled goat anti-rat (Invitrogen, A21094).

Validation

All primary antibodies were validated by the manufacturers. Bands of correct molecular weights were detected from: anti-HA by Western blot analysis using a cell line, that expresses a recombinant HA-tagged protein; anti-INTS1, anti-INTS4, anti-INTS7, anti-INTS9, anti-INTS10, anti-INTS11, anti-INTS13, anti-INTS14, anti-β-actin, anti-Dynein IC by Western blot against total human cell lysate; anti-GST by Western blot of cells expressing GST-tagged proteins.

Eukaryotic cell lines

Policy information about cell lines

Cell line source(s)	HEK293T (ATCC), HeLa (ATCC), Sf9 (Thermo Fisher), High Five (Thermo Fisher)
Authentication	Cell lines were not authenticated.
Mycoplasma contamination	Cells were tested negative for mycoplasma infection using PCR.

Commonly misidentified lines
(See ICLAC register)

No commonly misidentified lines were used in this study.
