# Supplementary material for: INTS10–INTS13–INTS14 form a functional module of Integrator that binds nucleic acids and the cleavage module
Source: Nat Commun. 2020 Jul 9;11:3422. doi: 10.1038/s41467-020-17232-2 (PMC7347597; doi:10.1038/s41467-020-17232-2)
Supplement: Supplementary file 4 — Description of Additional Supplementary Files [file 41467_2020_17232_MOESM4_ESM.pdf]

Description of Additional Supplementary Files

File name: Supplementary Data 1
Description: List of crosslinks identified by XL-MS for the INTS13-INTS14 complex.

File name: Supplementary Data 2
Description: List of crosslinks identified by XL-MS for the INTS13 CMBM-INTS4-INTS9- INTS11 complex.
