# Supplementary material for: INTS10–INTS13–INTS14 form a functional module of Integrator that binds nucleic acids and the cleavage module
Source: Nat Commun. 2020 Jul 9;11:3422. doi: 10.1038/s41467-020-17232-2 (PMC7347597; doi:10.1038/s41467-020-17232-2)
Supplement: Supplementary file 2 — Peer Review File [file 41467_2020_17232_MOESM2_ESM.pdf]

**Editorial Note:** This manuscript has been previously reviewed at another journal that is not operating a transparent peer review scheme. This document only contains reviewer comments and rebuttal letters for versions considered at *Nature Communications.*

REVIEWERS' COMMENTS:

Reviewer #1 (Remarks to the Author):

In this revised manuscript, Sabath and colleagues have refined their analysis on a newly identified structural/functional module of the Integrator complex.

In its original version, this manuscript presented compelling biochemical and structural evidence of the INTS13/14 heterodimer. However, the data proposing INTS10 as a third component of the module were lacking some in vivo validation (which is now offered in the form of a native nuclear extract gel fractionation).

In the second part of their manuscript, the authors identify an intriguing similarity between INTS13/14 and Ku70/80, which propels their study of nucleic acid binding abilities of their module. The data presented suggest affinity for RNA, both single stranded and in the form of stem loops. The authors then propose a role for RNA binding in that it would help directing the cleavage module at the proper cutting site for nascent UsnRNAs. This hypothesis is further supported by data suggesting that the C terminal domain of INTS13 bridges the interaction with the 4-9-11 catalytic module. The in vivo data on the actual contribution of INTS13, INTS14 or INTS10 to UsnRNA processing (probing both endogenous loci and reporter constructs) partly supports their hypothesis, however the phenotype is somewhat modest and points to an overall ancillary role in UsnRNA biogenesis.

In this improved version, the authors explore additional roles of Integrator and the potential contribution of the INTS13/14 module. They present data obtained with an HIV-promoter reporter system as a proxy to probe Pol2 pausing. Depletion of the module components results in a significant but moderate effect on pausing/elongation. Overall, these data still suggest that the main function of the module could lie elsewhere.
Nonetheless, I think the overall results are interesting and provide significant advance to our understanding of this evolutionarily conserved nuclear complex.

MINOR POINTS
In the discussion, the authors point out, rightfully, that their findings about the cleavage module may hold implications for how INTS13 regulates myeloid differentiation. They further speculate that INTS13/14 and INTS11 might not be that functionally separated after all and that could be an antibody bias. While it is true that antibodies may preferentially recognize particular isoforms of a protein or even interfere with protein-protein interactions, data from that study (Mol Cell 2018) indicate that INTS13 antibodies do co-precipitate copious amounts of INTS11/9/4 when used on a total nuclear extract. Why would that be different in a fractionated extract?

Reviewer #2 (Remarks to the Author):

The authors have investigated the structures and interactions between subunits 10, 13 and 14 of the Integrator Complex (INTS10, 13 and 14).
They have shown that INTS13 and INTS14 co-immunoprecipitate from HEK293 and insect cells after ectopic expression of tagged proteins as a stochiometric and stable heterodimer. They also carried out size exclusion chromatography, which confirms that a complex of INTS10/13/14 exists in nuclear extract.
The crystal structure of INTS13/14 solved at 2.5 A indicates that although the two proteins do not have a high degree of primary sequence similarity, they have similar domain structures and form an

intertwined pseudo-symmetric heterodimer. Sequence conservation suggests that this structure is conserved in all metazoans that express INTS13 and INTS14. In addition, the interlinked structure suggests that folding and dimerization are linked.
Crosslinking mass spectrometry confirms that the conformation from crystallography is present in solution and shows that the complex oligomerizes. In addition, mutation of predicted interaction regions disrupt complex formation.

The authors have also shown that INTS13/14 co-immunoprecipitates with INTS10 and that this heterotrimer is stable through purification. Mutational analysis indicates that INTS10 interacts with INTS14, in line with previous yeast 2-hybrid analyses of Drosophila INTS10 and INTS14 homologues. The authors define the VWA domain of INTS14 as necessary and sufficient for interaction with INTS10.

Together this data defines INTS10/13/14 as a subcomplex of Integrator.
As INTS13/14 has some homology to Ku70/Ku80, which binds nucleic acids, the authors have investigated the ability of INTS13/14 and INTS10/13/14 to interact with nucleic acid using EMSA and fluorescence polarization. INTS13/14 binds ssRNA best, whereas INTS10/13/14 has higher affinity for nucleic acid and prefers dsRNA.

Both INTS13/14 and INTS10/13/14 co-immunoprecipitate INTS4/9/11, which they term the cleavage module and the authors present evidence that interaction is through the C-term of INTS13 and that the full INTS4/9/11 module is needed for interaction.

Finally, the authors have assayed the effect of knockdown (KD) of INTS subunits on expression of renilla luciferase (rLuc) downstream from a U7 3' box, with the rationale that cleavage at the 3' box will down regulate rLuc expression. In line with this, KD of INTS11 causes a massive increase in rLuc expression. KD of INTS10, 13 or 14 has a more modest effect. In addition, deletion of the C-term of INTS13 appears to reduce the ability of INTS13 to complement INTS13 KD. In a very nice HIV-1 TAR-mediated termination assay that the authors have set up, KD of INTS10 or 13 or 14 causes a modest increase in expression of a downstream gene, presumably due to increased readthrough.

The structural studies are very well done and the structure for INTS13/14 is new and interesting. In addition, the nucleic acid binding studies support the idea that INTS10/13/14 could help Integrator interact with RNA, although there does not seem to be that much specificity.

Unfortunately, the study doesn't really succeed in going much beyond the structures and interactions to shed light on the molecular functions of the subunits. In addition, I think I would only conclude from the experiments in Figure 6b-g that the function of INTS10, 13 or 14 is not very important for 3' processing and cleavage as the effect is so much less that INTS11 KD. Knockdown of INTS7, another non-catalytic subunit, has a much bigger effect in this assay.

The evidence that the RNA-binding module recruits the cleavage module to 3' boxes is therefore not strong as loss of this module has only a modest effect on cleavage of snRNA gene transcripts. The authors do note that KD of INTS10 or 13 or 14 has a relatively stronger effect in their HIV-1 premature termination system. However, also in the HIV-1 assay, KD of INTS10, 13 or 14 has much less effect than KD of INTS7, suggesting that these subunits are playing a minor role in both systems or that KD is not getting rid of these subunits from the stably-associated complexes or their role in the tested systems just isn't terribly important in cell lines. It is interesting that KD of INTS11 also doesn't have a big effect in this assay. As KD of INTS11 (and INTS9) also has less of an effect than KD of INTS7 and 8 in the Drosophila MtnA system, perhaps cleavage is not the main mechanism inhibiting transcription in these systems as suggested by the authors. Relevant to this, KD of INTS10 or 13 or 14 has very little effect in the Drosophila MtnA system. Perhaps an unbiased analysis of the effect of KD of these subunits on transcription and RNA processing or a more profound knockdown using degrons or a more in vivo system would help to determine whether these subunits play a major role in gene expression.

Other comments

Lines 10-12. The authors should make it clear that the studies on Integrator causing termination of paused RNAPII that they cite were in Drosophila.

Figure 3a. Is INTS11 modified in fractions 16/18, where the authors say the INTS10/13/14 complex is? Looks to me that there are many subcomplexes eg INTS1/4/13/14 and INTS10/11(modified)/13/14. Also, INTS7 looks like it is running faster in fractions 16/18.

Figure 7a. Should there really be scissors on the diagram? KD of INTS11 has a much more modest effect here than in the U7 assay and the authors say themselves that they think that Integrator is doing something besides just cleaving the RNA.

Figure 7C. This is a very nice summary diagram but I have the suspicion that INTS10/13/14 plays a rather bigger role in some other function(s) in the cell.
Typos/references

Abstract. Line 5: form not from

Page 18, line 4. Is 25 the right ref here?

Throughout. Lots of stray commas and the English needs a bit of editing.

## Response to Reviewers' Comments:

*We thank the reviewers for considering our revised manuscript and for their constructive comments in both reviewing rounds that helped us to improve our paper.*

**Reviewer #1:**

In this revised manuscript, Sabath and colleagues have refined their analysis on a newly identified structural/functional module of the Integrator complex.

In its original version, this manuscript presented compelling biochemical and structural evidence of the INTS13/14 heterodimer. However, the data proposing INTS10 as a third component of the module were lacking some in vivo validation (which is now offered in the form of a native nuclear extract gel fractionation).

In the second part of their manuscript, the authors identify an intriguing similarity between INTS13/14 and Ku70/80, which propels their study of nucleic acid binding abilities of their module. The data presented suggest affinity for RNA, both single stranded and in the form of stem loops. The authors then propose a role for RNA binding in that it would help directing the cleavage module at the proper cutting site for nascent UsnRNAs. This hypothesis is further supported by data suggesting that the C terminal domain of INTS13 bridges the interaction with the 4-9-11 catalytic module. The in vivo data on the actual contribution of INTS13, INTS14 or INTS10 to UsnRNA processing (probing both endogenous loci and reporter constructs) partly supports their hypothesis, however the phenotype is somewhat modest and points to an overall ancillary role in UsnRNA biogenesis.

In this improved version, the authors explore additional roles of Integrator and the potential contribution of the INTS13/14 module. They present data obtained with an HIV-promoter reporter system as a proxy to probe Pol2 pausing. Depletion of the module components results in a significant but moderate effect on pausing/elongation. Overall, these data still suggest that the main function of the module could lie elsewhere.

Nonetheless, I think the overall results are interesting and provide significant advance to our understanding of this evolutionarily conserved nuclear complex.

MINOR POINTS

In the discussion, the authors point out, rightfully, that their findings about the cleavage module may hold implications for how INTS13 regulates myeloid differentiation. They further speculate that INTS13/14 and INTS11 might not be that functionally separated after all and that could be an antibody bias. While it is true that antibodies may preferentially recognize particular isoforms of a protein or even interfere with protein-protein interactions, data from that study (Mol Cell 2018) indicate that INTS13 antibodies do co-precipitate copious amounts of INTS11/9/4 when used on a total nuclear extract. Why would that be different in a fractionated extract?

*→ The INTS10-13-14 module does not only bind the INTS4-9-11 cleavage module, but it also interacts via the INTS10-axis with the core subunits of INT, which in turn also bind the cleavage module. Thus, the identification of INTS4-9-11 in that IP-MS experiment (Barbieri et al. Mol. Cell 2018, Fig. 6A) would be expected even if the INTS13 antibody impairs the direct binding of the cleavage module to the INTS13 CMBM. Indeed, in agreement with our hypothesis, the enrichment factors of INTS4-9-11 in that study are lower than several other INTS that are not directly binding to INTS13.*

*However, we agree that we have not proven this point in our study, and thus we weakened our statement in the discussion to underline its hypothetical nature.*

**Reviewer #2:**

The authors have investigated the structures and interactions between subunits 10, 13 and 14 of the Integrator Complex (INTS10, 13 and 14). They have shown that INTS13 and INTS14 co-immunoprecipitate

from HEK293 and insect cells after ectopic expression of tagged proteins as a stochiometric and stable heterodimer. They also carried out size exclusion chromatography, which confirms that a complex of INTS10/13/14 exists in nuclear extract. The crystal structure of INTS13/14 solved at 2.5 A indicates that although the two proteins do not have a high degree of primary sequence similarity, they have similar domain structures and form an intertwined pseudo-symmetric heterodimer. Sequence conservation suggests that this structure is conserved in all metazoans that express INTS13 and INTS14. In addition, the interlinked structure suggests that folding and dimerization are linked. Crosslinking mass spectrometry confirms that the conformation from crystallography is present in solution and shows that the complex oligomerizes. In addition, mutation of predicted interaction regions disrupt complex formation.

The authors have also shown that INTS13/14 co-immunoprecipitates with INTS10 and that this heterotrimer is stable through purification. Mutational analysis indicates that INTS10 interacts with INTS14, in line with previous yeast 2-hybrid analyses of Drosophila INTS10 and INTS14 homologues. The authors define the VWA domain of INTS14 as necessary and sufficient for interaction with INTS10. Together this data defines INTS10/13/14 as a subcomplex of Integrator.

As INTS13/14 has some homology to Ku70/Ku80, which binds nucleic acids, the authors have investigated the ability of INTS13/14 and INTS10/13/14 to interact with nucleic acid using EMSA and fluorescence polarization. INTS13/14 binds ssRNA best, whereas INTS10/13/14 has higher affinity for nucleic acid and prefers dsRNA.

Both INTS13/14 and INTS10/13/14 co-immunoprecipitate INTS4/9/11, which they term the cleavage module and the authors present evidence that interaction is through the C-term of INTS13 and that the full INTS4/9/11 module is needed for interaction.

Finally, the authors have assayed the effect of knockdown (KD) of INTS subunits on expression of renilla luciferase (rLuc) downstream from a U7 3' box, with the rationale that cleavage at the 3' box will down regulate rLuc expression. In line with this, KD of INTS11 causes a massive increase in rLuc expression. KD of INTS10, 13 or 14 has a more modest effect. In addition, deletion of the C-term of INTS13 appears to reduce the ability of INTS13 to complement INTS13 KD. In a very nice HIV-1 TAR-mediated termination assay that the authors have set up, KD of INTS10 or 13 or 14 causes a modest increase in expression of a downstream gene, presumably due to increased readthrough.

The structural studies are very well done and the structure for INTS13/14 is new and interesting. In addition, the nucleic acid binding studies support the idea that INTS10/13/14 could help Integrator interact with RNA, although there does not seem to be that much specificity.

Unfortunately, the study doesn't really succeed in going much beyond the structures and interactions to shed light on the molecular functions of the subunits. In addition, I think I would only conclude from the experiments in Figure 6b-g that the function of INTS10, 13 or 14 is not very important for 3' processing and cleavage as the effect is so much less that INTS11 KD. Knockdown of INTS7, another non-catalytic subunit, has a much bigger effect in this assay.

The evidence that the RNA-binding module recruits the cleavage module to 3' boxes is therefore not strong as loss of this module has only a modest effect on cleavage of snRNA gene transcripts. The authors do note that KD of INTS10 or 13 or 14 has a relatively stronger effect in their HIV-1 premature termination system. However, also in the HIV-1 assay, KD of INTS10, 13 or 14 has much less effect than KD of INTS7, suggesting that these subunits are playing a minor role in both systems or that KD is not getting rid of these subunits from the stably-associated complexes or their role in the tested systems just isn't terribly important in cell lines. It is interesting that KD of INTS11 also doesn't have a big effect in this assay. As KD of INTS11 (and INTS9) also has less of an effect than KD of INTS7 and 8 in the Drosophila MtnA system, perhaps cleavage is not the main mechanism inhibiting transcription in these systems as suggested by the authors. Relevant to this, KD of INTS10 or 13 or 14 has very little effect in the Drosophila MtnA system. Perhaps an unbiased analysis of the effect of KD of these subunits on transcription and RNA processing or a more profound knockdown using degrons or a more in vivo system would help to determine whether these subunits play a major role in gene expression.

*→ We maintain that it may be misleading to infer the biological importance of any given factor of a pathway from its quantitative effect in an assay. All the proteins we study here are essential for metazoan embryonic development and we show for two functions of INT (ncRNA processing and mRNA transcription termination after pausing) that their depletion impairs these functions. Furthermore, we describe molecular roles for a previously uncharacterized module of INT, and determined the structure of two of its subunits.*

*We agree that a genome wide study on the direct targets of INTS10, INTS13 and INTS14 and delineating how their specific regulation is brought about would be very interesting. However, we think that such a study does lie not within the scope of this current manuscript and would be an endeavor worthy of an independent publication.*

Other comments

Lines 10-12. The authors should make it clear that the studies on Integrator causing termination of paused RNAPII that they cite were in Drosophila.

*→ We added this information as requested.*

Figure 3a. Is INTS11 modified in fractions 16/18, where the authors say the INTS10/13/14 complex is? Looks to me that there are many subcomplexes eg INTS1/4/13/14 and INTS10/11(modified)/13/14. Also, INTS7 looks like it is running faster in fractions 16/18.

*→ We agree with the reviewer that there seem to be potentially more INT subcomplexes in cells. However, the point, that we address with this experiment is to show that the INTS10-INTS13-INTS14 module does indeed form in the nucleus, and that this subcomplex is separable from the holo-INT complex. Given that the nuclear INTS10-INTS13-INTS14 module migrates in a separate peak that behaves analogously to the purified, recombinant INTS10-INTS13-INTS14 module on a size exclusion column, our data indicates that the complex does indeed exist in cells.*

*INTS11 always shows two bands on Western blots, which are both reduced upon siRNA-mediated depletion. Since INTS11 has several isoforms and could potentially also be modified post-translationally, we do currently not know what the nature of these two bands is. In order to keep our manuscript focused we chose not to speculate on these aspects, for which we currently have no data and which are leading away from the purpose of this experiment.*

Figure 7a. Should there really be scissors on the diagram? KD of INTS11 has a much more modest effect here than in the U7 assay and the authors say themselves that they think that Integrator is doing something besides just cleaving the RNA.

*→ Two publications on transcription termination after pausing (Tatomer 2019, Elrod 2019) have shown that INTS11 endonucleolytic activity is required for termination. INT cleavage may not be the only function of INT that leads to RNAPII termination, but it is definitely an important part of it. Analogously, CPF (cleavage and polyadenylation factor) mediated transcript cleavage is important for faithful RNAPII termination close to poly-adenylation sites, however this is not the only reaction required for RNAPII transcription termination. Therefore, the depiction of scissors is in keeping with literature data.*

Figure 7C. This is a very nice summary diagram but I have the suspicion that INTS10/13/14 plays a rather bigger role in some other function(s) in the cell.

*→ We agree that it is possible that the module has additional functions. The purpose of this figure is to summarize the molecular insight that we have gained so far on the Integrator complex architecture with specific focus on the newly characterized DNA/RNA binding module, while avoiding potentially misleading or speculative indications. To make this clearer we now state in the figure legend that it is a "summary model".*

Typos/references

Abstract. Line 5: form not from

→ *Thank you, this has now been corrected.*

Page 18, line 4. Is 25 the right ref here?

→ *Thank you, we removed the reference.*

Throughout. Lots of stray commas and the English needs a bit of editing.

→ *We have edited the text and removed several commas.*
